# Supplementary material for: Prevalence and factors associated with contraceptive use among sexually active adolescent girls in 25 sub-Saharan African countries
Source: PLoS One. 2024 Feb 28;19(2):e0297411. doi: 10.1371/journal.pone.0297411 (PMC10901330; doi:10.1371/journal.pone.0297411)
Supplement: S1 File — (DOCX) [file pone.0297411.s001.docx]

**Sample by Country and Contraceptive Use Results:**

**Frequencies**

| **Notes** | | |
| --- | --- | --- |
| Output Created | | 09-MAY-2023 12:07:00 |
| Comments | |  |
| Input | Data | C:\Users\Admi\Documents\Documents\DOCUMENTS\DHS DATA SETS (SUB SAHARAN AFRICA\DHS data (Sub-Saharan 25 Countries COMBINED_IR (aged 15-19 & sexually active selected ONly USED Variables.sav |
|  | Active Dataset | DataSet1 |
|  | Filter | <none> |
|  | Weight | WGT |
|  | Split File | <none> |
|  | N of Rows in Working Data File | 16546 |
| Missing Value Handling | Definition of Missing | User-defined missing values are treated as missing. |
|  | Cases Used | Statistics are based on all cases with valid data. |
| Syntax | | FREQUENCIES VARIABLES=V000  /ORDER=ANALYSIS. |
| Resources | Processor Time | 00:00:00.02 |
|  | Elapsed Time | 00:00:00.02 |

| **Statistics** | | |
| --- | --- | --- |
| Country code and phase | | |
| N | Valid | 16442 |
|  | Missing | 0 |

| **Country code and phase** | | | | | |
| --- | --- | --- | --- | --- | --- |
|  | | Frequency | Percent | Valid Percent | Cumulative Percent |
| Valid | AO7 | 961 | 5.8 | 5.8 | 5.8 |
|  | BJ7 | 743 | 4.5 | 4.5 | 10.4 |
|  | BU7 | 230 | 1.4 | 1.4 | 11.8 |
|  | CD6 | 1075 | 6.5 | 6.5 | 18.3 |
|  | ET7 | 504 | 3.1 | 3.1 | 21.4 |
|  | GA6 | 612 | 3.7 | 3.7 | 25.1 |
|  | GH6 | 228 | 1.4 | 1.4 | 26.5 |
|  | GM7 | 255 | 1.5 | 1.5 | 28.0 |
|  | GN7 | 493 | 3.0 | 3.0 | 31.0 |
|  | KE6 | 311 | 1.9 | 1.9 | 32.9 |
|  | LB7 | 498 | 3.0 | 3.0 | 35.9 |
|  | LS6 | 182 | 1.1 | 1.1 | 37.0 |
|  | MD7 | 1389 | 8.4 | 8.4 | 45.5 |
|  | ML7 | 789 | 4.8 | 4.8 | 50.3 |
|  | MW7 | 1180 | 7.2 | 7.2 | 57.5 |
|  | NG7 | 1913 | 11.6 | 11.6 | 69.1 |
|  | NI6 | 696 | 4.2 | 4.2 | 73.3 |
|  | NM6 | 248 | 1.5 | 1.5 | 74.8 |
|  | RW7 | 112 | .7 | .7 | 75.5 |
|  | SL7 | 972 | 5.9 | 5.9 | 81.4 |
|  | TD6 | 1164 | 7.1 | 7.1 | 88.5 |
|  | TZ7 | 700 | 4.3 | 4.3 | 92.8 |
|  | ZA7 | 241 | 1.5 | 1.5 | 94.2 |
|  | ZM7 | 566 | 3.4 | 3.4 | 97.7 |
|  | ZW7 | 384 | 2.3 | 2.3 | 100.0 |
|  | Total | 16442 | 100.0 | 100.0 |  |

**Prevalence of modern contraceptive:**

**Crosstabs**

| **Notes** | | |
| --- | --- | --- |
| Output Created | | 11-MAY-2023 15:30:50 |
| Comments | |  |
| Input | Data | C:\Users\Admi\Documents\Documents\DOCUMENTS\DHS DATA SETS (SUB SAHARAN AFRICA\DHS data (Sub-Saharan 25 Countries COMBINED_IR (aged 15-19 & sexually active selected ONly USED Variables.sav |
|  | Active Dataset | DataSet1 |
|  | Filter | <none> |
|  | Weight | WGT |
|  | Split File | <none> |
|  | N of Rows in Working Data File | 16546 |
| Missing Value Handling | Definition of Missing | User-defined missing values are treated as missing. |
|  | Cases Used | Statistics for each table are based on all the cases with valid data in the specified range(s) for all variables in each table. |
| Syntax | | CROSSTABS  /TABLES=V000 BY CONTRaceptiveUSeTypeREC  /FORMAT=AVALUE TABLES  /CELLS=COUNT ROW  /COUNT ROUND CELL. |
| Resources | Processor Time | 00:00:00.05 |
|  | Elapsed Time | 00:00:00.07 |
|  | Dimensions Requested | 2 |
|  | Cells Available | 524245 |

[DataSet1] C:\Users\Admi\Documents\Documents\DOCUMENTS\DHS DATA SETS (SUB SAHARAN AFRICA\DHS data (Sub-Saharan 25 Countries COMBINED_IR (aged 15-19 & sexually active selected ONly USED Variables.sav

| **Case Processing Summary** | | | | | | |
| --- | --- | --- | --- | --- | --- | --- |
|  | Cases | | | | | |
|  | Valid | | Missing | | Total | |
|  | N | Percent | N | Percent | N | Percent |
| Country code and phase * CONTRaceptive USe Type REC | 16443 | 100.0% | 0 | 0.0% | 16442.314 | 100.0% |

| **Country code and phase * CONTRaceptive USe Type REC Crosstabulation** | | | | | | |
| --- | --- | --- | --- | --- | --- | --- |
|  | | | CONTRaceptive USe Type REC | | | Total |
|  |  |  | No method | Traditional method | Modern method |  |
| Country code and phase | AO7 | Count | 806 | 9 | 145 | 960 |
|  |  | % within Country code and phase | 84.0% | 0.9% | 15.1% | 100.0% |
|  | BJ7 | Count | 626 | 16 | 101 | 743 |
|  |  | % within Country code and phase | 84.3% | 2.2% | 13.6% | 100.0% |
|  | BU7 | Count | 171 | 5 | 53 | 229 |
|  |  | % within Country code and phase | 74.7% | 2.2% | 23.1% | 100.0% |
|  | CD6 | Count | 786 | 157 | 133 | 1076 |
|  |  | % within Country code and phase | 73.0% | 14.6% | 12.4% | 100.0% |
|  | ET7 | Count | 304 | 1 | 198 | 503 |
|  |  | % within Country code and phase | 60.4% | 0.2% | 39.4% | 100.0% |
|  | GA6 | Count | 286 | 61 | 266 | 613 |
|  |  | % within Country code and phase | 46.7% | 10.0% | 43.4% | 100.0% |
|  | GH6 | Count | 137 | 22 | 69 | 228 |
|  |  | % within Country code and phase | 60.1% | 9.6% | 30.3% | 100.0% |
|  | GM7 | Count | 231 | 3 | 21 | 255 |
|  |  | % within Country code and phase | 90.6% | 1.2% | 8.2% | 100.0% |
|  | GN7 | Count | 386 | 5 | 102 | 493 |
|  |  | % within Country code and phase | 78.3% | 1.0% | 20.7% | 100.0% |
|  | KE6 | Count | 164 | 11 | 135 | 310 |
|  |  | % within Country code and phase | 52.9% | 3.5% | 43.5% | 100.0% |
|  | LB7 | Count | 319 | 4 | 175 | 498 |
|  |  | % within Country code and phase | 64.1% | 0.8% | 35.1% | 100.0% |
|  | LS6 | Count | 83 | 2 | 98 | 183 |
|  |  | % within Country code and phase | 45.4% | 1.1% | 53.6% | 100.0% |
|  | MD7 | Count | 750 | 78 | 560 | 1388 |
|  |  | % within Country code and phase | 54.0% | 5.6% | 40.3% | 100.0% |
|  | ML7 | Count | 671 | 10 | 107 | 788 |
|  |  | % within Country code and phase | 85.2% | 1.3% | 13.6% | 100.0% |
|  | MW7 | Count | 707 | 12 | 462 | 1181 |
|  |  | % within Country code and phase | 59.9% | 1.0% | 39.1% | 100.0% |
|  | NG7 | Count | 1775 | 34 | 104 | 1913 |
|  |  | % within Country code and phase | 92.8% | 1.8% | 5.4% | 100.0% |
|  | NI6 | Count | 635 | 11 | 50 | 696 |
|  |  | % within Country code and phase | 91.2% | 1.6% | 7.2% | 100.0% |
|  | NM6 | Count | 92 | 6 | 150 | 248 |
|  |  | % within Country code and phase | 37.1% | 2.4% | 60.5% | 100.0% |
|  | RW7 | Count | 66 | 1 | 45 | 112 |
|  |  | % within Country code and phase | 58.9% | 0.9% | 40.2% | 100.0% |
|  | SL7 | Count | 497 | 4 | 470 | 971 |
|  |  | % within Country code and phase | 51.2% | 0.4% | 48.4% | 100.0% |
|  | TD6 | Count | 1109 | 9 | 46 | 1164 |
|  |  | % within Country code and phase | 95.3% | 0.8% | 4.0% | 100.0% |
|  | TZ7 | Count | 538 | 21 | 141 | 700 |
|  |  | % within Country code and phase | 76.9% | 3.0% | 20.1% | 100.0% |
|  | ZA7 | Count | 97 | 0 | 144 | 241 |
|  |  | % within Country code and phase | 40.2% | 0.0% | 59.8% | 100.0% |
|  | ZM7 | Count | 343 | 3 | 220 | 566 |
|  |  | % within Country code and phase | 60.6% | 0.5% | 38.9% | 100.0% |
|  | ZW7 | Count | 205 | 4 | 175 | 384 |
|  |  | % within Country code and phase | 53.4% | 1.0% | 45.6% | 100.0% |
| Total | | Count | 11784 | 489 | 4170 | 16443 |
|  |  | % within Country code and phase | 71.7% | 3.0% | 25.4% | 100.0% |
