# Supplementary material for: Prevalence and factors associated with contraceptive use among sexually active adolescent girls in 25 sub-Saharan African countries
Source: PLoS One. 2024 Feb 28;19(2):e0297411. doi: 10.1371/journal.pone.0297411 (PMC10901330; doi:10.1371/journal.pone.0297411)
Supplement: S3 File — (DOCX) [file pone.0297411.s003.docx]

VIF Collinearity Statistics Results

COMPUTE WGT=V005/1000000.

WEIGHT BY WGT.

REGRESSION

/MISSING LISTWISE

/STATISTICS COEFF OUTS R ANOVA COLLIN TOL

/CRITERIA=PIN(.05) POUT(.10)

/NOORIGIN

/DEPENDENT CONTRaceptiveUseTypeForBinaryREC

/METHOD=ENTER V106 MAritalSTatusREC IDEalNo.OfChildrenREc TOtalCHildrenEverBornREc V228 V384A

V384B V384C V850B V781 V190 V025.

**Regression**

| **Notes** | | |
| --- | --- | --- |
| Output Created | | 27-MAY-2023 05:32:35 |
| Comments | |  |
| Input | Data | C:\Users\Admi\Documents\DOCUMENTS\DHS DATA SETS (SUB SAHARAN AFRICA\DHS data (Sub-Saharan 25 Countries COMBINED_IR (aged 15-19 & sexually active selected ONly USED Variables.sav |
|  | Active Dataset | DataSet1 |
|  | Filter | <none> |
|  | Weight | WGT |
|  | Split File | <none> |
|  | N of Rows in Working Data File | 16546 |
| Missing Value Handling | Definition of Missing | User-defined missing values are treated as missing. |
|  | Cases Used | Statistics are based on cases with no missing values for any variable used. |
| Syntax | | REGRESSION  /MISSING LISTWISE  /STATISTICS COEFF OUTS R ANOVA COLLIN TOL  /CRITERIA=PIN(.05) POUT(.10)  /NOORIGIN  /DEPENDENT CONTRaceptiveUseTypeForBinaryREC  /METHOD=ENTER V106 MAritalSTatusREC IDEalNo.OfChildrenREc TOtalCHildrenEverBornREc V228 V384A  V384B V384C V850B V781 V190 V025. |
| Resources | Processor Time | 00:00:00.22 |
|  | Elapsed Time | 00:00:01.63 |
|  | Memory Required | 12656 bytes |
|  | Additional Memory Required for Residual Plots | 0 bytes |

| **Variables Entered/Removed^a^** | | | |
| --- | --- | --- | --- |
| Model | Variables Entered | Variables Removed | Method |
| 1 | Type of place of residence, Respondent can ask partner to use a condom, TOtal CHildren Ever Born REc, Heard family planning on radio last few months, Ever had a terminated pregnancy, IDEal No. Of Children REc, Heard family planning in newspaper/magazine last few months, Ever been tested for HIV, Heard family planning on TV last few months, Highest educational level, Wealth index combined^b^ | . | Enter |
| a. Dependent Variable: CONTRaceptive Use Type For Binary REC | | | |
| b. All requested variables entered. | | | |

| **Model Summary** | | | | |
| --- | --- | --- | --- | --- |
| Model | R | R Square | Adjusted R Square | Std. Error of the Estimate |
| 1 | .440^a^ | .193 | .192 | .396 |
| a. Predictors: (Constant), Type of place of residence, Respondent can ask partner to use a condom, TOtal CHildren Ever Born REc, Heard family planning on radio last few months, Ever had a terminated pregnancy, IDEal No. Of Children REc, Heard family planning in newspaper/magazine last few months, Ever been tested for HIV, Heard family planning on TV last few months, Highest educational level, Wealth index combined | | | | |

| **ANOVA^a^** | | | | | | |
| --- | --- | --- | --- | --- | --- | --- |
| Model | | Sum of Squares | df | Mean Square | F | Sig. |
| 1 | Regression | 273.934 | 11 | 24.903 | 158.653 | .000^b^ |
|  | Residual | 1142.965 | 7282 | .157 |  |  |
|  | Total | 1416.899 | 7293 |  |  |  |
| a. Dependent Variable: CONTRaceptive Use Type For Binary REC | | | | | | |
| b. Predictors: (Constant), Type of place of residence, Respondent can ask partner to use a condom, TOtal CHildren Ever Born REc, Heard family planning on radio last few months, Ever had a terminated pregnancy, IDEal No. Of Children REc, Heard family planning in newspaper/magazine last few months, Ever been tested for HIV, Heard family planning on TV last few months, Highest educational level, Wealth index combined | | | | | | |

| **Coefficients^a^** | | | | | | | | |
| --- | --- | --- | --- | --- | --- | --- | --- | --- |
| Model | | Unstandardized Coefficients | | Standardized Coefficients | t | Sig. | Collinearity Statistics | |
|  |  | B | Std. Error | Beta |  |  | Tolerance | VIF |
| 1 | (Constant) | .024 | .037 |  | .649 | .516 |  |  |
|  | Highest educational level | .091 | .007 | .157 | 13.160 | .000 | .781 | 1.280 |
|  | IDEal No. Of Children REc | -.089 | .008 | -.129 | -11.223 | .000 | .837 | 1.194 |
|  | TOtal CHildren Ever Born REc | .176 | .007 | .267 | 24.882 | .000 | .961 | 1.040 |
|  | Ever had a terminated pregnancy | -.045 | .017 | -.028 | -2.684 | .007 | .987 | 1.013 |
|  | Heard family planning on radio last few months | .048 | .011 | .047 | 4.181 | .000 | .864 | 1.157 |
|  | Heard family planning on TV last few months | -.029 | .018 | -.019 | -1.637 | .102 | .786 | 1.272 |
|  | Heard family planning in newspaper/magazine last few months | .038 | .027 | .016 | 1.422 | .155 | .891 | 1.122 |
|  | Respondent can ask partner to use a condom | .000 | .002 | -.002 | -.147 | .883 | .992 | 1.008 |
|  | Ever been tested for HIV | .153 | .010 | .171 | 14.697 | .000 | .815 | 1.228 |
|  | Wealth index combined | .016 | .004 | .047 | 3.791 | .000 | .734 | 1.362 |
|  | Type of place of residence | .063 | .013 | .060 | 4.894 | .000 | .728 | 1.373 |
| a. Dependent Variable: CONTRaceptive Use Type For Binary REC | | | | | | | | |

| **Collinearity Diagnostics^a^** | | | | | | | | | | | | | | | |
| --- | --- | --- | --- | --- | --- | --- | --- | --- | --- | --- | --- | --- | --- | --- | --- |
| Model | Dimension | Eigenvalue | Condition Index | Variance Proportions | | | | | | | | | | | |
|  |  |  |  | (Constant) | Highest educational level | IDEal No. Of Children REc | TOtal CHildren Ever Born REc | Ever had a terminated pregnancy | Heard family planning on radio last few months | Heard family planning on TV last few months | Heard family planning in newspaper/magazine last few months | Respondent can ask partner to use a condom | Ever been tested for HIV | Wealth index combined | Type of place of residence |
| 1 | 1 | 6.283 | 1.000 | .00 | .01 | .00 | .01 | .00 | .01 | .00 | .00 | .01 | .01 | .00 | .00 |
|  | 2 | 1.290 | 2.207 | .00 | .00 | .00 | .01 | .00 | .06 | .18 | .26 | .03 | .00 | .00 | .00 |
|  | 3 | .919 | 2.615 | .00 | .00 | .00 | .01 | .92 | .00 | .00 | .00 | .02 | .00 | .00 | .00 |
|  | 4 | .768 | 2.860 | .00 | .01 | .00 | .01 | .02 | .00 | .01 | .08 | .77 | .05 | .00 | .00 |
|  | 5 | .717 | 2.961 | .00 | .00 | .00 | .00 | .00 | .12 | .32 | .56 | .01 | .03 | .00 | .00 |
|  | 6 | .569 | 3.322 | .00 | .03 | .00 | .00 | .01 | .46 | .22 | .04 | .03 | .21 | .00 | .00 |
|  | 7 | .512 | 3.503 | .00 | .01 | .01 | .07 | .00 | .33 | .16 | .05 | .11 | .30 | .00 | .00 |
|  | 8 | .447 | 3.749 | .00 | .09 | .00 | .66 | .04 | .01 | .00 | .01 | .01 | .06 | .03 | .00 |
|  | 9 | .272 | 4.809 | .00 | .63 | .02 | .19 | .00 | .01 | .03 | .00 | .00 | .24 | .00 | .01 |
|  | 10 | .158 | 6.299 | .00 | .12 | .01 | .03 | .00 | .00 | .07 | .00 | .00 | .00 | .65 | .04 |
|  | 11 | .053 | 10.919 | .01 | .03 | .75 | .00 | .00 | .01 | .01 | .00 | .00 | .05 | .06 | .25 |
|  | 12 | .012 | 23.213 | .99 | .08 | .21 | .01 | .00 | .00 | .01 | .00 | .01 | .04 | .25 | .70 |
| a. Dependent Variable: CONTRaceptive Use Type For Binary REC | | | | | | | | | | | | | | | |
