# Supplementary material for: Prevalence and factors associated with contraceptive use among sexually active adolescent girls in 25 sub-Saharan African countries
Source: PLoS One. 2024 Feb 28;19(2):e0297411. doi: 10.1371/journal.pone.0297411 (PMC10901330; doi:10.1371/journal.pone.0297411)
Supplement: S4 File — (DOCX) [file pone.0297411.s004.docx]

**DESCRIPTIVE STATISTICS - WEIGHTED FREQUENCIES & CHI-SQUARE RESULTS:**

**Frequencies:**

| **Notes** | | |
| --- | --- | --- |
| Output Created | | 09-MAY-2023 11:59:03 |
| Comments | |  |
| Input | Data | C:\Users\Admi\Documents\Documents\DOCUMENTS\DHS DATA SETS (SUB SAHARAN AFRICA\DHS data (Sub-Saharan 25 Countries COMBINED_IR (aged 15-19 & sexually active selected ONly USED Variables.sav |
|  | Active Dataset | DataSet1 |
|  | Filter | <none> |
|  | Weight | WGT |
|  | Split File | <none> |
|  | N of Rows in Working Data File | 16546 |
| Missing Value Handling | Definition of Missing | User-defined missing values are treated as missing. |
|  | Cases Used | Statistics are based on all cases with valid data. |
| Syntax | | FREQUENCIES VARIABLES=V106 MAritalSTatusREC V714 IDEalNo.OfChildrenREc TOtalCHildrenEverBornREc  V228 V384A V384B V384C NUMberofSexPARtnerREC V850B V781 V190 V025  /ORDER=ANALYSIS. |
| Resources | Processor Time | 00:00:00.05 |
|  | Elapsed Time | 00:00:00.05 |

| **Statistics** | | | | | | | | | | | | | | | |
| --- | --- | --- | --- | --- | --- | --- | --- | --- | --- | --- | --- | --- | --- | --- | --- |
|  | | Highest educational level | MArital STatus REC | Respondent currently working | IDEal No. Of Children REc | TOtal CHildren Ever Born REc | Ever had a terminated pregnancy | Heard family planning on radio last few months | Heard family planning on TV last few months | Heard family planning in newspaper/magazine last few months | NUMber of Sex PARtner REC | Respondent can ask partner to use a condom | Ever been tested for HIV | Wealth index combined | Type of place of residence |
| N | Valid | 16441 | 16442 | 16414 | 15775 | 16442 | 16436 | 16434 | 16438 | 16442 | 16441 | 9306 | 13813 | 16442 | 16442 |
|  | Missing | 1 | 0 | 29 | 668 | 0 | 6 | 8 | 5 | 1 | 1 | 7136 | 2629 | 0 | 0 |

**Frequency Table**

| **Highest educational level** | | | | | |
| --- | --- | --- | --- | --- | --- |
|  | | Frequency | Percent | Valid Percent | Cumulative Percent |
| Valid | No education | 4732 | 28.8 | 28.8 | 28.8 |
|  | Primary | 5743 | 34.9 | 34.9 | 63.7 |
|  | Secondary | 5854 | 35.6 | 35.6 | 99.3 |
|  | Higher | 113 | .7 | .7 | 100.0 |
|  | Total | 16441 | 100.0 | 100.0 |  |
| Missing | 9 | 1 | .0 |  |  |
| Total | | 16442 | 100.0 |  |  |

| **MArital STatus REC** | | | | | |
| --- | --- | --- | --- | --- | --- |
|  | | Frequency | Percent | Valid Percent | Cumulative Percent |
| Valid | Never in union | 5469 | 33.3 | 33.3 | 33.3 |
|  | Ever married | 10973 | 66.7 | 66.7 | 100.0 |
|  | Total | 16442 | 100.0 | 100.0 |  |

| **Respondent currently working** | | | | | |
| --- | --- | --- | --- | --- | --- |
|  | | Frequency | Percent | Valid Percent | Cumulative Percent |
| Valid | No | 9235 | 56.2 | 56.3 | 56.3 |
|  | Yes | 7178 | 43.7 | 43.7 | 100.0 |
|  | Total | 16414 | 99.8 | 100.0 |  |
| Missing | 9 | 29 | .2 |  |  |
| Total | | 16442 | 100.0 |  |  |

| **IDEal No. Of Children REc** | | | | | |
| --- | --- | --- | --- | --- | --- |
|  | | Frequency | Percent | Valid Percent | Cumulative Percent |
| Valid | 0-2 | 1998 | 12.1 | 12.7 | 12.7 |
|  | 3-5 | 8764 | 53.3 | 55.6 | 68.2 |
|  | 6+ | 5013 | 30.5 | 31.8 | 100.0 |
|  | Total | 15775 | 95.9 | 100.0 |  |
| Missing | System | 668 | 4.1 |  |  |
| Total | | 16442 | 100.0 |  |  |

| **TOtal CHildren Ever Born REc** | | | | | |
| --- | --- | --- | --- | --- | --- |
|  | | Frequency | Percent | Valid Percent | Cumulative Percent |
| Valid | None | 9307 | 56.6 | 56.6 | 56.6 |
|  | 1 | 5747 | 35.0 | 35.0 | 91.6 |
|  | 2 or more | 1388 | 8.4 | 8.4 | 100.0 |
|  | Total | 16442 | 100.0 | 100.0 |  |

| **Ever had a terminated pregnancy** | | | | | |
| --- | --- | --- | --- | --- | --- |
|  | | Frequency | Percent | Valid Percent | Cumulative Percent |
| Valid | No | 15295 | 93.0 | 93.1 | 93.1 |
|  | Yes | 1141 | 6.9 | 6.9 | 100.0 |
|  | Total | 16436 | 100.0 | 100.0 |  |
| Missing | 9 | 6 | .0 |  |  |
| Total | | 16442 | 100.0 |  |  |

| **Heard family planning on radio last few months** | | | | | |
| --- | --- | --- | --- | --- | --- |
|  | | Frequency | Percent | Valid Percent | Cumulative Percent |
| Valid | No | 12177 | 74.1 | 74.1 | 74.1 |
|  | Yes | 4258 | 25.9 | 25.9 | 100.0 |
|  | Total | 16434 | 100.0 | 100.0 |  |
| Missing | 9 | 8 | .0 |  |  |
| Total | | 16442 | 100.0 |  |  |

| **Heard family planning on TV last few months** | | | | | |
| --- | --- | --- | --- | --- | --- |
|  | | Frequency | Percent | Valid Percent | Cumulative Percent |
| Valid | No | 14534 | 88.4 | 88.4 | 88.4 |
|  | Yes | 1904 | 11.6 | 11.6 | 100.0 |
|  | Total | 16438 | 100.0 | 100.0 |  |
| Missing | 9 | 5 | .0 |  |  |
| Total | | 16442 | 100.0 |  |  |

| **Heard family planning in newspaper/magazine last few months** | | | | | |
| --- | --- | --- | --- | --- | --- |
|  | | Frequency | Percent | Valid Percent | Cumulative Percent |
| Valid | No | 15628 | 95.0 | 95.0 | 95.0 |
|  | Yes | 814 | 5.0 | 5.0 | 100.0 |
|  | Total | 16442 | 100.0 | 100.0 |  |
| Missing | 9 | 1 | .0 |  |  |
| Total | | 16442 | 100.0 |  |  |

| **NUMber of Sex PARtner REC** | | | | | |
| --- | --- | --- | --- | --- | --- |
|  | | Frequency | Percent | Valid Percent | Cumulative Percent |
| Valid | 1 | 14819 | 90.1 | 90.1 | 90.1 |
|  | 2 or more | 1622 | 9.9 | 9.9 | 100.0 |
|  | Total | 16441 | 100.0 | 100.0 |  |
| Missing | System | 1 | .0 |  |  |
| Total | | 16442 | 100.0 |  |  |

| **Respondent can ask partner to use a condom** | | | | | |
| --- | --- | --- | --- | --- | --- |
|  | | Frequency | Percent | Valid Percent | Cumulative Percent |
| Valid | No | 4558 | 27.7 | 49.0 | 49.0 |
|  | Yes | 4183 | 25.4 | 44.9 | 93.9 |
|  | Don't know/not sure/depends | 565 | 3.4 | 6.1 | 100.0 |
|  | Total | 9306 | 56.6 | 100.0 |  |
| Missing | 9 | 14 | .1 |  |  |
|  | System | 7123 | 43.3 |  |  |
|  | Total | 7136 | 43.4 |  |  |
| Total | | 16442 | 100.0 |  |  |

| **Ever been tested for HIV** | | | | | |
| --- | --- | --- | --- | --- | --- |
|  | | Frequency | Percent | Valid Percent | Cumulative Percent |
| Valid | No | 9134 | 55.6 | 66.1 | 66.1 |
|  | Yes | 4679 | 28.5 | 33.9 | 100.0 |
|  | Total | 13813 | 84.0 | 100.0 |  |
| Missing | 9 | 16 | .1 |  |  |
|  | System | 2613 | 15.9 |  |  |
|  | Total | 2629 | 16.0 |  |  |
| Total | | 16442 | 100.0 |  |  |

| **Wealth index combined** | | | | | |
| --- | --- | --- | --- | --- | --- |
|  | | Frequency | Percent | Valid Percent | Cumulative Percent |
| Valid | Poorest | 3665 | 22.3 | 22.3 | 22.3 |
|  | Poorer | 3789 | 23.0 | 23.0 | 45.3 |
|  | Middle | 3560 | 21.6 | 21.6 | 67.0 |
|  | Richer | 3137 | 19.1 | 19.1 | 86.1 |
|  | Richest | 2291 | 13.9 | 13.9 | 100.0 |
|  | Total | 16442 | 100.0 | 100.0 |  |

| **Type of place of residence** | | | | | |
| --- | --- | --- | --- | --- | --- |
|  | | Frequency | Percent | Valid Percent | Cumulative Percent |
| Valid | Urban | 5042 | 30.7 | 30.7 | 30.7 |
|  | Rural | 11400 | 69.3 | 69.3 | 100.0 |
|  | Total | 16442 | 100.0 | 100.0 |  |

**CHI-SQUARE RESULTS:**

**Crosstabs**

| **Notes** | | |
| --- | --- | --- |
| Output Created | | 09-MAY-2023 11:44:28 |
| Comments | |  |
| Input | Data | C:\Users\Admi\Documents\Documents\DOCUMENTS\DHS DATA SETS (SUB SAHARAN AFRICA\DHS data (Sub-Saharan 25 Countries COMBINED_IR (aged 15-19 & sexually active selected ONly USED Variables.sav |
|  | Active Dataset | DataSet1 |
|  | Filter | <none> |
|  | Weight | WGT |
|  | Split File | <none> |
|  | N of Rows in Working Data File | 16546 |
| Missing Value Handling | Definition of Missing | User-defined missing values are treated as missing. |
|  | Cases Used | Statistics for each table are based on all the cases with valid data in the specified range(s) for all variables in each table. |
| Syntax | | CROSSTABS  /TABLES=V106 MAritalSTatusREC V714 IDEalNo.OfChildrenREc TOtalCHildrenEverBornREc V228 V384A  V384B V384C NUMberofSexPARtnerREC V850B V781 V190 V025 BY CONTRaceptiveUSeTypeREC  /FORMAT=AVALUE TABLES  /STATISTICS=CHISQ  /CELLS=COUNT ROW  /COUNT ROUND CELL. |
| Resources | Processor Time | 00:00:00.06 |
|  | Elapsed Time | 00:00:00.06 |
|  | Dimensions Requested | 2 |
|  | Cells Available | 524245 |

| **Case Processing Summary** | | | | | | |
| --- | --- | --- | --- | --- | --- | --- |
|  | Cases | | | | | |
|  | Valid | | Missing | | Total | |
|  | N | Percent | N | Percent | N | Percent |
| Highest educational level * CONTRaceptive USe Type REC | 16443 | 100.0% | 0 | 0.0% | 16442.314 | 100.0% |
| MArital STatus REC * CONTRaceptive USe Type REC | 16442^a^ | 100.0% | .314 | 0.0% | 16442.314 | 100.0% |
| Respondent currently working * CONTRaceptive USe Type REC | 16414^a^ | 99.8% | 28.314 | 0.2% | 16442.314 | 100.0% |
| IDEal No. Of Children REc * CONTRaceptive USe Type REC | 15775^a^ | 95.9% | 667.314 | 4.1% | 16442.314 | 100.0% |
| TOtal CHildren Ever Born REc * CONTRaceptive USe Type REC | 16443^a^ | 100.0% | 0 | 0.0% | 16442.314 | 100.0% |
| Ever had a terminated pregnancy * CONTRaceptive USe Type REC | 16435^a^ | 100.0% | 7.314 | 0.0% | 16442.314 | 100.0% |
| Heard family planning on radio last few months * CONTRaceptive USe Type REC | 16435^a^ | 100.0% | 7.314 | 0.0% | 16442.314 | 100.0% |
| Heard family planning on TV last few months * CONTRaceptive USe Type REC | 16436^a^ | 100.0% | 6.314 | 0.0% | 16442.314 | 100.0% |
| Heard family planning in newspaper/magazine last few months * CONTRaceptive USe Type REC | 16442^a^ | 100.0% | .314 | 0.0% | 16442.314 | 100.0% |
| NUMber of Sex PARtner REC * CONTRaceptive USe Type REC | 16441^a^ | 100.0% | 1.314 | 0.0% | 16442.314 | 100.0% |
| Respondent can ask partner to use a condom * CONTRaceptive USe Type REC | 9306^a^ | 56.6% | 7136.314 | 43.4% | 16442.314 | 100.0% |
| Ever been tested for HIV * CONTRaceptive USe Type REC | 13813^a^ | 84.0% | 2629.314 | 16.0% | 16442.314 | 100.0% |
| Wealth index combined * CONTRaceptive USe Type REC | 16442^a^ | 100.0% | .314 | 0.0% | 16442.314 | 100.0% |
| Type of place of residence * CONTRaceptive USe Type REC | 16442^a^ | 100.0% | .314 | 0.0% | 16442.314 | 100.0% |
| a. Number of valid cases is different from the total count in the crosstabulation table because the cell counts have been rounded. | | | | | | |

**Highest educational level * CONTRaceptive USe Type REC**

| **Crosstab** | | | | | | |
| --- | --- | --- | --- | --- | --- | --- |
|  | | | CONTRaceptive USe Type REC | | | Total |
|  |  |  | No method | Traditional method | Modern method |  |
| Highest educational level | No education | Count | 4309 | 34 | 389 | 4732 |
|  |  | % within Highest educational level | 91.1% | 0.7% | 8.2% | 100.0% |
|  | Primary | Count | 4062 | 125 | 1557 | 5744 |
|  |  | % within Highest educational level | 70.7% | 2.2% | 27.1% | 100.0% |
|  | Secondary | Count | 3358 | 326 | 2170 | 5854 |
|  |  | % within Highest educational level | 57.4% | 5.6% | 37.1% | 100.0% |
|  | Higher | Count | 55 | 3 | 55 | 113 |
|  |  | % within Highest educational level | 48.7% | 2.7% | 48.7% | 100.0% |
| Total | | Count | 11784 | 488 | 4171 | 16443 |
|  |  | % within Highest educational level | 71.7% | 3.0% | 25.4% | 100.0% |

| **Chi-Square Tests** | | | |
| --- | --- | --- | --- |
|  | Value | df | Asymptotic Significance (2-sided) |
| Pearson Chi-Square | 1546.322^a^ | 6 | <.001 |
| Likelihood Ratio | 1701.892 | 6 | <.001 |
| Linear-by-Linear Association | 1363.417 | 1 | <.001 |
| N of Valid Cases | 16443 |  |  |
| a. 1 cells (8.3%) have expected count less than 5. The minimum expected count is 3.35. | | | |

**MArital STatus REC * CONTRaceptive USe Type REC**

| **Crosstab** | | | | | | |
| --- | --- | --- | --- | --- | --- | --- |
|  | | | CONTRaceptive USe Type REC | | | Total |
|  |  |  | No method | Traditional method | Modern method |  |
| MArital STatus REC | Never in union | Count | 3079 | 291 | 2099 | 5469 |
|  |  | % within MArital STatus REC | 56.3% | 5.3% | 38.4% | 100.0% |
|  | Ever married | Count | 8705 | 196 | 2072 | 10973 |
|  |  | % within MArital STatus REC | 79.3% | 1.8% | 18.9% | 100.0% |
| Total | | Count | 11784 | 487 | 4171 | 16442 |
|  |  | % within MArital STatus REC | 71.7% | 3.0% | 25.4% | 100.0% |

| **Chi-Square Tests** | | | |
| --- | --- | --- | --- |
|  | Value | df | Asymptotic Significance (2-sided) |
| Pearson Chi-Square | 971.048^a^ | 2 | <.001 |
| Likelihood Ratio | 938.970 | 2 | <.001 |
| Linear-by-Linear Association | 873.191 | 1 | <.001 |
| N of Valid Cases | 16442 |  |  |
| a. 0 cells (0.0%) have expected count less than 5. The minimum expected count is 161.99. | | | |

**Respondent currently working * CONTRaceptive USe Type REC**

| **Crosstab** | | | | | | |
| --- | --- | --- | --- | --- | --- | --- |
|  | | | CONTRaceptive USe Type REC | | | Total |
|  |  |  | No method | Traditional method | Modern method |  |
| Respondent currently working | No | Count | 6622 | 271 | 2342 | 9235 |
|  |  | % within Respondent currently working | 71.7% | 2.9% | 25.4% | 100.0% |
|  | Yes | Count | 5135 | 216 | 1828 | 7179 |
|  |  | % within Respondent currently working | 71.5% | 3.0% | 25.5% | 100.0% |
| Total | | Count | 11757 | 487 | 4170 | 16414 |
|  |  | % within Respondent currently working | 71.6% | 3.0% | 25.4% | 100.0% |

| **Chi-Square Tests** | | | |
| --- | --- | --- | --- |
|  | Value | df | Asymptotic Significance (2-sided) |
| Pearson Chi-Square | .110^a^ | 2 | .947 |
| Likelihood Ratio | .110 | 2 | .947 |
| Linear-by-Linear Association | .042 | 1 | .838 |
| N of Valid Cases | 16414 |  |  |
| a. 0 cells (0.0%) have expected count less than 5. The minimum expected count is 213.00. | | | |

**IDEal No. Of Children REc * CONTRaceptive USe Type REC**

| **Crosstab** | | | | | | |
| --- | --- | --- | --- | --- | --- | --- |
|  | | | CONTRaceptive USe Type REC | | | Total |
|  |  |  | No method | Traditional method | Modern method |  |
| IDEal No. Of Children REc | 0-2 | Count | 1137 | 59 | 801 | 1997 |
|  |  | % within IDEal No. Of Children REc | 56.9% | 3.0% | 40.1% | 100.0% |
|  | 3-5 | Count | 5635 | 328 | 2802 | 8765 |
|  |  | % within IDEal No. Of Children REc | 64.3% | 3.7% | 32.0% | 100.0% |
|  | 6+ | Count | 4402 | 94 | 517 | 5013 |
|  |  | % within IDEal No. Of Children REc | 87.8% | 1.9% | 10.3% | 100.0% |
| Total | | Count | 11174 | 481 | 4120 | 15775 |
|  |  | % within IDEal No. Of Children REc | 70.8% | 3.0% | 26.1% | 100.0% |

| **Chi-Square Tests** | | | |
| --- | --- | --- | --- |
|  | Value | df | Asymptotic Significance (2-sided) |
| Pearson Chi-Square | 1091.981^a^ | 4 | <.001 |
| Likelihood Ratio | 1201.096 | 4 | <.001 |
| Linear-by-Linear Association | 977.868 | 1 | <.001 |
| N of Valid Cases | 15775 |  |  |
| a. 0 cells (0.0%) have expected count less than 5. The minimum expected count is 60.89. | | | |

**TOtal CHildren Ever Born REc * CONTRaceptive USe Type REC**

| **Crosstab** | | | | | | |
| --- | --- | --- | --- | --- | --- | --- |
|  | | | CONTRaceptive USe Type REC | | | Total |
|  |  |  | No method | Traditional method | Modern method |  |
| TOtal CHildren Ever Born REc | None | Count | 7029 | 307 | 1971 | 9307 |
|  |  | % within TOtal CHildren Ever Born REc | 75.5% | 3.3% | 21.2% | 100.0% |
|  | 1 | Count | 3696 | 151 | 1901 | 5748 |
|  |  | % within TOtal CHildren Ever Born REc | 64.3% | 2.6% | 33.1% | 100.0% |
|  | 2 or more | Count | 1059 | 30 | 299 | 1388 |
|  |  | % within TOtal CHildren Ever Born REc | 76.3% | 2.2% | 21.5% | 100.0% |
| Total | | Count | 11784 | 488 | 4171 | 16443 |
|  |  | % within TOtal CHildren Ever Born REc | 71.7% | 3.0% | 25.4% | 100.0% |

| **Chi-Square Tests** | | | |
| --- | --- | --- | --- |
|  | Value | df | Asymptotic Significance (2-sided) |
| Pearson Chi-Square | 282.653^a^ | 4 | <.001 |
| Likelihood Ratio | 276.526 | 4 | <.001 |
| Linear-by-Linear Association | 76.672 | 1 | <.001 |
| N of Valid Cases | 16443 |  |  |
| a. 0 cells (0.0%) have expected count less than 5. The minimum expected count is 41.19. | | | |

**Ever had a terminated pregnancy * CONTRaceptive USe Type REC**

| **Crosstab** | | | | | | |
| --- | --- | --- | --- | --- | --- | --- |
|  | | | CONTRaceptive USe Type REC | | | Total |
|  |  |  | No method | Traditional method | Modern method |  |
| Ever had a terminated pregnancy | No | Count | 10900 | 439 | 3955 | 15294 |
|  |  | % within Ever had a terminated pregnancy | 71.3% | 2.9% | 25.9% | 100.0% |
|  | Yes | Count | 878 | 47 | 216 | 1141 |
|  |  | % within Ever had a terminated pregnancy | 77.0% | 4.1% | 18.9% | 100.0% |
| Total | | Count | 11778 | 486 | 4171 | 16435 |
|  |  | % within Ever had a terminated pregnancy | 71.7% | 3.0% | 25.4% | 100.0% |

| **Chi-Square Tests** | | | |
| --- | --- | --- | --- |
|  | Value | df | Asymptotic Significance (2-sided) |
| Pearson Chi-Square | 30.467^a^ | 2 | <.001 |
| Likelihood Ratio | 31.605 | 2 | <.001 |
| Linear-by-Linear Association | 22.323 | 1 | <.001 |
| N of Valid Cases | 16435 |  |  |
| a. 0 cells (0.0%) have expected count less than 5. The minimum expected count is 33.74. | | | |

**Heard family planning on radio last few months * CONTRaceptive USe Type REC**

| **Crosstab** | | | | | | |
| --- | --- | --- | --- | --- | --- | --- |
|  | | | CONTRaceptive USe Type REC | | | Total |
|  |  |  | No method | Traditional method | Modern method |  |
| Heard family planning on radio last few months | No | Count | 9013 | 340 | 2824 | 12177 |
|  |  | % within Heard family planning on radio last few months | 74.0% | 2.8% | 23.2% | 100.0% |
|  | Yes | Count | 2768 | 147 | 1343 | 4258 |
|  |  | % within Heard family planning on radio last few months | 65.0% | 3.5% | 31.5% | 100.0% |
| Total | | Count | 11781 | 487 | 4167 | 16435 |
|  |  | % within Heard family planning on radio last few months | 71.7% | 3.0% | 25.4% | 100.0% |

| **Chi-Square Tests** | | | |
| --- | --- | --- | --- |
|  | Value | df | Asymptotic Significance (2-sided) |
| Pearson Chi-Square | 127.107^a^ | 2 | <.001 |
| Likelihood Ratio | 123.717 | 2 | <.001 |
| Linear-by-Linear Association | 125.783 | 1 | <.001 |
| N of Valid Cases | 16435 |  |  |
| a. 0 cells (0.0%) have expected count less than 5. The minimum expected count is 126.17. | | | |

**Heard family planning on TV last few months * CONTRaceptive USe Type REC**

| **Crosstab** | | | | | | |
| --- | --- | --- | --- | --- | --- | --- |
|  | | | CONTRaceptive USe Type REC | | | Total |
|  |  |  | No method | Traditional method | Modern method |  |
| Heard family planning on TV last few months | No | Count | 10636 | 390 | 3507 | 14533 |
|  |  | % within Heard family planning on TV last few months | 73.2% | 2.7% | 24.1% | 100.0% |
|  | Yes | Count | 1147 | 97 | 659 | 1903 |
|  |  | % within Heard family planning on TV last few months | 60.3% | 5.1% | 34.6% | 100.0% |
| Total | | Count | 11783 | 487 | 4166 | 16436 |
|  |  | % within Heard family planning on TV last few months | 71.7% | 3.0% | 25.3% | 100.0% |

| **Chi-Square Tests** | | | |
| --- | --- | --- | --- |
|  | Value | df | Asymptotic Significance (2-sided) |
| Pearson Chi-Square | 145.381^a^ | 2 | <.001 |
| Likelihood Ratio | 135.667 | 2 | <.001 |
| Linear-by-Linear Association | 122.036 | 1 | <.001 |
| N of Valid Cases | 16436 |  |  |
| a. 0 cells (0.0%) have expected count less than 5. The minimum expected count is 56.39. | | | |

**Heard family planning in newspaper/magazine last few months * CONTRaceptive USe Type REC**

| **Crosstab** | | | | | | |
| --- | --- | --- | --- | --- | --- | --- |
|  | | | CONTRaceptive USe Type REC | | | Total |
|  |  |  | No method | Traditional method | Modern method |  |
| Heard family planning in newspaper/magazine last few months | No | Count | 11339 | 450 | 3839 | 15628 |
|  |  | % within Heard family planning in newspaper/magazine last few months | 72.6% | 2.9% | 24.6% | 100.0% |
|  | Yes | Count | 445 | 37 | 332 | 814 |
|  |  | % within Heard family planning in newspaper/magazine last few months | 54.7% | 4.5% | 40.8% | 100.0% |
| Total | | Count | 11784 | 487 | 4171 | 16442 |
|  |  | % within Heard family planning in newspaper/magazine last few months | 71.7% | 3.0% | 25.4% | 100.0% |

| **Chi-Square Tests** | | | |
| --- | --- | --- | --- |
|  | Value | df | Asymptotic Significance (2-sided) |
| Pearson Chi-Square | 122.044^a^ | 2 | <.001 |
| Likelihood Ratio | 112.077 | 2 | <.001 |
| Linear-by-Linear Association | 119.059 | 1 | <.001 |
| N of Valid Cases | 16442 |  |  |
| a. 0 cells (0.0%) have expected count less than 5. The minimum expected count is 24.11. | | | |

**NUMber of Sex PARtner REC * CONTRaceptive USe Type REC**

| **Crosstab** | | | | | | |
| --- | --- | --- | --- | --- | --- | --- |
|  | | | CONTRaceptive USe Type REC | | | Total |
|  |  |  | No method | Traditional method | Modern method |  |
| NUMber of Sex PARtner REC | 1 | Count | 10743 | 405 | 3671 | 14819 |
|  |  | % within NUMber of Sex PARtner REC | 72.5% | 2.7% | 24.8% | 100.0% |
|  | 2 or more | Count | 1040 | 82 | 500 | 1622 |
|  |  | % within NUMber of Sex PARtner REC | 64.1% | 5.1% | 30.8% | 100.0% |
| Total | | Count | 11783 | 487 | 4171 | 16441 |
|  |  | % within NUMber of Sex PARtner REC | 71.7% | 3.0% | 25.4% | 100.0% |

| **Chi-Square Tests** | | | |
| --- | --- | --- | --- |
|  | Value | df | Asymptotic Significance (2-sided) |
| Pearson Chi-Square | 62.056^a^ | 2 | <.001 |
| Likelihood Ratio | 57.146 | 2 | <.001 |
| Linear-by-Linear Association | 40.265 | 1 | <.001 |
| N of Valid Cases | 16441 |  |  |
| a. 0 cells (0.0%) have expected count less than 5. The minimum expected count is 48.05. | | | |

**Respondent can ask partner to use a condom * CONTRaceptive USe Type REC**

| **Crosstab** | | | | | | |
| --- | --- | --- | --- | --- | --- | --- |
|  | | | CONTRaceptive USe Type REC | | | Total |
|  |  |  | No method | Traditional method | Modern method |  |
| Respondent can ask partner to use a condom | No | Count | 3953 | 56 | 549 | 4558 |
|  |  | % within Respondent can ask partner to use a condom | 86.7% | 1.2% | 12.0% | 100.0% |
|  | Yes | Count | 2844 | 105 | 1233 | 4182 |
|  |  | % within Respondent can ask partner to use a condom | 68.0% | 2.5% | 29.5% | 100.0% |
|  | Don't know/not sure/depends | Count | 493 | 8 | 65 | 566 |
|  |  | % within Respondent can ask partner to use a condom | 87.1% | 1.4% | 11.5% | 100.0% |
| Total | | Count | 7290 | 169 | 1847 | 9306 |
|  |  | % within Respondent can ask partner to use a condom | 78.3% | 1.8% | 19.8% | 100.0% |

| **Chi-Square Tests** | | | |
| --- | --- | --- | --- |
|  | Value | df | Asymptotic Significance (2-sided) |
| Pearson Chi-Square | 479.179^a^ | 4 | <.001 |
| Likelihood Ratio | 481.258 | 4 | <.001 |
| Linear-by-Linear Association | .171 | 1 | .679 |
| N of Valid Cases | 9306 |  |  |
| a. 0 cells (0.0%) have expected count less than 5. The minimum expected count is 10.28. | | | |

**Ever been tested for HIV * CONTRaceptive USe Type REC**

| **Crosstab** | | | | | | |
| --- | --- | --- | --- | --- | --- | --- |
|  | | | CONTRaceptive USe Type REC | | | Total |
|  |  |  | No method | Traditional method | Modern method |  |
| Ever been tested for HIV | No | Count | 6852 | 319 | 1963 | 9134 |
|  |  | % within Ever been tested for HIV | 75.0% | 3.5% | 21.5% | 100.0% |
|  | Yes | Count | 2605 | 113 | 1961 | 4679 |
|  |  | % within Ever been tested for HIV | 55.7% | 2.4% | 41.9% | 100.0% |
| Total | | Count | 9457 | 432 | 3924 | 13813 |
|  |  | % within Ever been tested for HIV | 68.5% | 3.1% | 28.4% | 100.0% |

| **Chi-Square Tests** | | | |
| --- | --- | --- | --- |
|  | Value | df | Asymptotic Significance (2-sided) |
| Pearson Chi-Square | 634.681^a^ | 2 | <.001 |
| Likelihood Ratio | 616.683 | 2 | <.001 |
| Linear-by-Linear Association | 605.154 | 1 | <.001 |
| N of Valid Cases | 13813 |  |  |
| a. 0 cells (0.0%) have expected count less than 5. The minimum expected count is 146.34. | | | |

**Wealth index combined * CONTRaceptive USe Type REC**

| **Crosstab** | | | | | | |
| --- | --- | --- | --- | --- | --- | --- |
|  | | | CONTRaceptive USe Type REC | | | Total |
|  |  |  | No method | Traditional method | Modern method |  |
| Wealth index combined | Poorest | Count | 2886 | 74 | 706 | 3666 |
|  |  | % within Wealth index combined | 78.7% | 2.0% | 19.3% | 100.0% |
|  | Poorer | Count | 2860 | 78 | 851 | 3789 |
|  |  | % within Wealth index combined | 75.5% | 2.1% | 22.5% | 100.0% |
|  | Middle | Count | 2540 | 117 | 902 | 3559 |
|  |  | % within Wealth index combined | 71.4% | 3.3% | 25.3% | 100.0% |
|  | Richer | Count | 2110 | 112 | 915 | 3137 |
|  |  | % within Wealth index combined | 67.3% | 3.6% | 29.2% | 100.0% |
|  | Richest | Count | 1388 | 106 | 797 | 2291 |
|  |  | % within Wealth index combined | 60.6% | 4.6% | 34.8% | 100.0% |
| Total | | Count | 11784 | 487 | 4171 | 16442 |
|  |  | % within Wealth index combined | 71.7% | 3.0% | 25.4% | 100.0% |

| **Chi-Square Tests** | | | |
| --- | --- | --- | --- |
|  | Value | df | Asymptotic Significance (2-sided) |
| Pearson Chi-Square | 293.628^a^ | 8 | <.001 |
| Likelihood Ratio | 290.501 | 8 | <.001 |
| Linear-by-Linear Association | 257.094 | 1 | <.001 |
| N of Valid Cases | 16442 |  |  |
| a. 0 cells (0.0%) have expected count less than 5. The minimum expected count is 67.86. | | | |

**Type of place of residence * CONTRaceptive USe Type REC**

| **Crosstab** | | | | | | |
| --- | --- | --- | --- | --- | --- | --- |
|  | | | CONTRaceptive USe Type REC | | | Total |
|  |  |  | No method | Traditional method | Modern method |  |
| Type of place of residence | Urban | Count | 3181 | 224 | 1637 | 5042 |
|  |  | % within Type of place of residence | 63.1% | 4.4% | 32.5% | 100.0% |
|  | Rural | Count | 8603 | 263 | 2534 | 11400 |
|  |  | % within Type of place of residence | 75.5% | 2.3% | 22.2% | 100.0% |
| Total | | Count | 11784 | 487 | 4171 | 16442 |
|  |  | % within Type of place of residence | 71.7% | 3.0% | 25.4% | 100.0% |

| **Chi-Square Tests** | | | |
| --- | --- | --- | --- |
|  | Value | df | Asymptotic Significance (2-sided) |
| Pearson Chi-Square | 273.006^a^ | 2 | <.001 |
| Likelihood Ratio | 264.895 | 2 | <.001 |
| Linear-by-Linear Association | 236.465 | 1 | <.001 |
| N of Valid Cases | 16442 |  |  |
| a. 0 cells (0.0%) have expected count less than 5. The minimum expected count is 149.34. | | | |
