# Supplementary material for: Prevalence and factors associated with contraceptive use among sexually active adolescent girls in 25 sub-Saharan African countries
Source: PLoS One. 2024 Feb 28;19(2):e0297411. doi: 10.1371/journal.pone.0297411 (PMC10901330; doi:10.1371/journal.pone.0297411)
Supplement: S5 File — (DOCX) [file pone.0297411.s005.docx]

**Logistic Regression: Adjusted Odds Ratio Result**

LOGISTIC REGRESSION VARIABLES CONTRaceptiveUseTypeForBinaryREC

/METHOD=ENTER Highesteducationallevel MAritalSTatusREC IDEalNo.OfChildrenREc TOtalCHildrenEverBornREc V228

V384A V384B V384C RESpondentCanAskPartnerToUSeCOndomREC V190 V025 V781

/CONTRAST (Highesteducationallevel)=Indicator(1)

/CONTRAST (MAritalSTatusREC)=Indicator(1)

/CONTRAST (IDEalNo.OfChildrenREc)=Indicator(1)

/CONTRAST (TOtalCHildrenEverBornREc)=Indicator(1)

/CONTRAST (V228)=Indicator(1)

/CONTRAST (V384A)=Indicator(1)

/CONTRAST (V384B)=Indicator(1)

/CONTRAST (V384C)=Indicator(1)

/CONTRAST (RESpondentCanAskPartnerToUSeCOndomREC)=Indicator(1)

/CONTRAST (V190)=Indicator(1)

/CONTRAST (V025)=Indicator(1)

/CONTRAST (V781)=Indicator(1)

/PRINT=GOODFIT CI(95)

/CRITERIA=PIN(0.05) POUT(0.10) ITERATE(20) CUT(0.5).

**Logistic Regression**

| **Notes** | | |
| --- | --- | --- |
| Output Created | | 26-MAY-2023 10:16:02 |
| Comments | |  |
| Input | Data | C:\Users\Admi\Documents\DOCUMENTS\DHS DATA SETS (SUB SAHARAN AFRICA\DHS data (Sub-Saharan 25 Countries COMBINED_IR (aged 15-19 & sexually active selected ONly USED Variables.sav |
|  | Active Dataset | DataSet1 |
|  | Filter | <none> |
|  | Weight | WGT |
|  | Split File | <none> |
|  | N of Rows in Working Data File | 16546 |
| Missing Value Handling | Definition of Missing | User-defined missing values are treated as missing |
| Syntax | | LOGISTIC REGRESSION VARIABLES CONTRaceptiveUseTypeForBinaryREC  /METHOD=ENTER  Highesteducationallevel MAritalSTatusREC IDEalNo.OfChildrenREc TOtalCHildrenEverBornREc V228  V384A V384B V384C RESpondentCanAskPartnerToUSeCOndomREC V190 V025 V781  /CONTRAST  (Highesteducationallevel)=Indicator(1)  /CONTRAST (MAritalSTatusREC)=Indicator(1)  /CONTRAST (IDEalNo.OfChildrenREc)=Indicator(1)  /CONTRAST (TOtalCHildrenEverBornREc)=Indicator(1)  /CONTRAST (V228)=Indicator(1)  /CONTRAST (V384A)=Indicator(1)  /CONTRAST (V384B)=Indicator(1)  /CONTRAST (V384C)=Indicator(1)  /CONTRAST (RESpondentCanAskPartnerToUSeCOndomREC)=Indicator(1)  /CONTRAST (V190)=Indicator(1)  /CONTRAST (V025)=Indicator(1)  /CONTRAST (V781)=Indicator(1)  /PRINT=GOODFIT CI(95)  /CRITERIA=PIN(0.05) POUT(0.10) ITERATE(20) CUT(0.5). |
| Resources | Processor Time | 00:00:00.29 |
|  | Elapsed Time | 00:00:00.44 |

| **Case Processing Summary** | | | |
| --- | --- | --- | --- |
| Unweighted Cases^a^ | | N | Percent |
| Selected Cases | Included in Analysis | 7472 | 45.2 |
|  | Missing Cases | 9074 | 54.8 |
|  | Total | 16546 | 100.0 |
| Unselected Cases | | 0 | .0 |
| Total | | 16546 | 100.0 |
| a. If weight is in effect, see classification table for the total number of cases. | | | |

| **Dependent Variable Encoding** | |
| --- | --- |
| Original Value | Internal Value |
| Used no method | 0 |
| Used any method | 1 |

| **Categorical Variables Codings** | | | | | | |
| --- | --- | --- | --- | --- | --- | --- |
|  | | Frequency | Parameter coding | | | |
|  |  |  | (1) | (2) | (3) | (4) |
| Wealth index combined | Poorest | 2021 | .000 | .000 | .000 | .000 |
|  | Poorer | 1836 | 1.000 | .000 | .000 | .000 |
|  | Middle | 1575 | .000 | 1.000 | .000 | .000 |
|  | Richer | 1221 | .000 | .000 | 1.000 | .000 |
|  | Richest | 819 | .000 | .000 | .000 | 1.000 |
| Highest educational level | No education | 2332 | .000 | .000 | .000 |  |
|  | Primary | 3224 | 1.000 | .000 | .000 |  |
|  | Secondary | 1898 | .000 | 1.000 | .000 |  |
|  | Higher | 18 | .000 | .000 | 1.000 |  |
| IDEal No. Of Children REc | 0-2 | 936 | .000 | .000 |  |  |
|  | 3-5 | 4012 | 1.000 | .000 |  |  |
|  | 6+ | 2524 | .000 | 1.000 |  |  |
| Respondent can ask partner to use a condom | No | 3300 | .000 | .000 |  |  |
|  | Yes | 3648 | 1.000 | .000 |  |  |
|  | Don't know/not sure/depends | 524 | .000 | 1.000 |  |  |
| TOtal CHildren Ever Born REc | None | 3150 | .000 | .000 |  |  |
|  | 1 | 3405 | 1.000 | .000 |  |  |
|  | 2 or more | 917 | .000 | 1.000 |  |  |
| Ever had a terminated pregnancy | No | 6845 | .000 |  |  |  |
|  | Yes | 627 | 1.000 |  |  |  |
| Heard family planning on radio last few months | No | 5599 | .000 |  |  |  |
|  | Yes | 1873 | 1.000 |  |  |  |
| Type of place of residence | Urban | 1793 | .000 |  |  |  |
|  | Rural | 5679 | 1.000 |  |  |  |
| Ever been tested for HIV | No | 4398 | .000 |  |  |  |
|  | Yes | 3074 | 1.000 |  |  |  |
| Heard family planning in newspaper/magazine last few months | No | 7234 | .000 |  |  |  |
|  | Yes | 238 | 1.000 |  |  |  |
| Heard family planning on TV last few months | No | 6785 | .000 |  |  |  |
|  | Yes | 687 | 1.000 |  |  |  |
| MArital STatus REC | Never in union | 5523 | .000 |  |  |  |
|  | Ever married | 11023 | 1.000 |  |  |  |

**Block 0: Beginning Block**

| **Classification Table^a,b^** | | | | | |
| --- | --- | --- | --- | --- | --- |
|  | Observed | | Predicted | | |
|  |  |  | CONTRaceptive Use Type For Binary REC | | Percentage Correct |
|  |  |  | Used no method | Used any method |  |
| Step 0 | CONTRaceptive Use Type For Binary REC | Used no method | 5369 | 0 | 100.0 |
|  |  | Used any method | 1925 | 0 | .0 |
|  | Overall Percentage | |  |  | 73.6 |
| a. Constant is included in the model. | | | | | |
| b. The cut value is .500 | | | | | |

| **Variables in the Equation** | | | | | | | |
| --- | --- | --- | --- | --- | --- | --- | --- |
|  | | B | S.E. | Wald | df | Sig. | Exp(B) |
| Step 0 | Constant | -1.026 | .027 | 1490.667 | 1 | .000 | .359 |

| **Variables not in the Equation** | | | | | |
| --- | --- | --- | --- | --- | --- |
|  | | | Score | df | Sig. |
| Step 0 | Variables | Highest educational level | 478.926 | 3 | .000 |
|  |  | Highest educational level(1) | 88.176 | 1 | .000 |
|  |  | Highest educational level(2) | 131.418 | 1 | .000 |
|  |  | Highest educational level(3) | 7.776 | 1 | .005 |
|  |  | IDEal No. Of Children REc | 415.782 | 2 | .000 |
|  |  | IDEal No. Of Children REc(1) | 178.029 | 1 | .000 |
|  |  | IDEal No. Of Children REc(2) | 405.887 | 1 | .000 |
|  |  | TOtal CHildren Ever Born REc | 942.748 | 2 | .000 |
|  |  | TOtal CHildren Ever Born REc(1) | 786.237 | 1 | .000 |
|  |  | TOtal CHildren Ever Born REc(2) | 5.369 | 1 | .020 |
|  |  | Ever had a terminated pregnancy(1) | 14.245 | 1 | .000 |
|  |  | Heard family planning on radio last few months(1) | 62.157 | 1 | .000 |
|  |  | Heard family planning on TV last few months(1) | 26.170 | 1 | .000 |
|  |  | Heard family planning in newspaper/magazine last few months(1) | 32.434 | 1 | .000 |
|  |  | Respondent can ask partner to use a condom | 312.227 | 2 | .000 |
|  |  | Respondent can ask partner to use a condom(1) | 309.981 | 1 | .000 |
|  |  | Respondent can ask partner to use a condom(2) | 36.756 | 1 | .000 |
|  |  | Ever been tested for HIV(1) | 608.442 | 1 | .000 |
|  |  | Wealth index combined | 39.853 | 4 | .000 |
|  |  | Wealth index combined(1) | .200 | 1 | .655 |
|  |  | Wealth index combined(2) | .004 | 1 | .953 |
|  |  | Wealth index combined(3) | .273 | 1 | .601 |
|  |  | Wealth index combined(4) | 30.165 | 1 | .000 |
|  |  | Type of place of residence(1) | 11.337 | 1 | .001 |
|  | Overall Statistics | | 1726.364 | 19 | .000 |

**Block 1: Method = Enter**

| **Omnibus Tests of Model Coefficients** | | | | |
| --- | --- | --- | --- | --- |
|  | | Chi-square | df | Sig. |
| Step 1 | Step | 1915.452 | 16 | .000 |
|  | Block | 1915.452 | 16 | .000 |
|  | Model | 1915.452 | 16 | .000 |

| **Model Summary** | | | |
| --- | --- | --- | --- |
| Step | -2 Log likelihood | Cox & Snell R Square | Nagelkerke R Square |
| 1 | 6503.075^a^ | .231 | .337 |
| a. Estimation terminated at iteration number 5 because parameter estimates changed by less than .001. | | | |

| **Hosmer and Lemeshow Test** | | | |
| --- | --- | --- | --- |
| Step | Chi-square | df | Sig. |
| 1 | 31.601 | 8 | .170 |

| **Contingency Table for Hosmer and Lemeshow Test** | | | | | | |
| --- | --- | --- | --- | --- | --- | --- |
|  | | CONTRaceptive Use Type For Binary REC = Used no method | | CONTRaceptive Use Type For Binary REC = Used any method | | Total |
|  |  | Observed | Expected | Observed | Expected |  |
| Step 1 | 1 | 717 | 721.609 | 18 | 12.867 | 734 |
|  | 2 | 695 | 698.031 | 34 | 31.012 | 729 |
|  | 3 | 656 | 672.573 | 73 | 55.889 | 728 |
|  | 4 | 645 | 638.559 | 74 | 80.717 | 719 |
|  | 5 | 603 | 604.616 | 113 | 111.319 | 716 |
|  | 6 | 590 | 563.461 | 140 | 166.989 | 730 |
|  | 7 | 537 | 500.070 | 192 | 228.846 | 729 |
|  | 8 | 372 | 409.107 | 355 | 317.328 | 726 |
|  | 9 | 309 | 323.115 | 421 | 407.408 | 731 |
|  | 10 | 244 | 237.560 | 506 | 512.544 | 750 |

| **Classification Table^a^** | | | | | |
| --- | --- | --- | --- | --- | --- |
|  | Observed | | Predicted | | |
|  |  |  | CONTRaceptive Use Type For Binary REC | | Percentage Correct |
|  |  |  | Used no method | Used any method |  |
| Step 1 | CONTRaceptive Use Type For Binary REC | Used no method | 4811 | 558 | 89.6 |
|  |  | Used any method | 989 | 936 | 48.6 |
|  | Overall Percentage | |  |  | 78.8 |
| a. The cut value is .500 | | | | | |

| **Variables in the Equation** | | | | | | | | | |
| --- | --- | --- | --- | --- | --- | --- | --- | --- | --- |
|  | | B | S.E. | Wald | df | Sig. | Exp(B) | 95% C.I.for EXP(B) | |
|  |  |  |  |  |  |  |  | Lower | Upper |
| Step 1^a^ | Highest educational level(1) | .975 | .093 | 110.087 | 1 | .000 | 2.650 | 2.209 | 3.180 |
|  | Highest educational level(2) | 1.190 | .103 | 132.217 | 1 | .000 | 3.286 | 2.683 | 4.025 |
|  | Highest educational level(3) | 2.562 | .568 | 20.369 | 1 | .000 | 7.965 | 6.261 | 9.446 |
|  | Marital STatus REC (1) | -1.092 | .036 | 917.278 | 1 | .000 | .336 | .313 | .360 |
|  | IDEal No. Of Children REc |  |  | 175.563 | 2 | .000 |  |  |  |
|  | IDEal No. Of Children REc(1) | -.192 | .088 | 4.756 | 1 | .029 | .825 | .694 | .981 |
|  | IDEal No. Of Children REc(2) | -1.221 | .110 | 123.012 | 1 | .000 | .295 | .238 | .366 |
|  | TOtal CHildren Ever Born REc |  |  | 716.224 | 2 | .000 |  |  |  |
|  | TOtal CHildren Ever Born REc(1) | 2.129 | .080 | 708.892 | 1 | .000 | 8.407 | 7.187 | 9.833 |
|  | TOtal CHildren Ever Born REc(2) | 1.933 | .109 | 314.296 | 1 | .000 | 6.911 | 5.581 | 8.558 |
|  | Ever had a terminated pregnancy(1) | -.114 | .118 | .927 | 1 | .336 | .892 | .708 | 1.125 |
|  | Heard family planning on radio last few months(1) | .225 | .072 | 9.671 | 1 | .002 | 1.253 | 1.087 | 1.444 |
|  | Heard family planning on TV last few months(1) | -.219 | .110 | 3.967 | 1 | .046 | .803 | .648 | .997 |
|  | Heard family planning in newspaper/magazine last few months(1) | .354 | .163 | 4.731 | 1 | .030 | 1.425 | 1.036 | 1.960 |
|  | RESpondent Can Ask Partner To USe COndom REC(1) | .534 | .066 | 65.868 | 1 | .000 | 1.705 | 1.499 | 1.940 |
|  | Wealth index combined |  |  | 38.930 | 4 | .000 |  |  |  |
|  | Wealth index combined(1) | .176 | .089 | 3.899 | 1 | .048 | 1.193 | 1.001 | 1.421 |
|  | Wealth index combined(2) | .200 | .094 | 4.528 | 1 | .033 | 1.221 | 1.016 | 1.467 |
|  | Wealth index combined(3) | .304 | .104 | 8.571 | 1 | .003 | 1.355 | 1.106 | 1.662 |
|  | Wealth index combined(4) | .802 | .130 | 38.237 | 1 | .000 | 2.230 | 1.729 | 2.875 |
|  | Type of place of residence(1) | .423 | .087 | 23.543 | 1 | .000 | 1.527 | 1.287 | 1.811 |
|  | Ever been tested for HIV(1) | .751 | .066 | 130.914 | 1 | .000 | 2.120 | 1.864 | 2.411 |
|  | Constant | -3.472 | .158 | 480.869 | 1 | .000 | .031 |  |  |
| a. Variable(s) entered on step 1: Highest Educational level, Marital STatus REC, IDEal No. Of Children REc, TOtal CHildren Ever Born REc, Ever had a terminated pregnancy, Heard family planning on radio last few months, Heard family planning on TV last few months, Heard family planning in newspaper/magazine last few months, RESpondent Can Ask Partner To USe COndom REC, Wealth index combined, Type of place of residence, Ever been tested for HIV. | | | | | | | | | |
