# Supplementary material for: Prevalence and factors associated with contraceptive use among sexually active adolescent girls in 25 sub-Saharan African countries
Source: PLoS One. 2024 Feb 28;19(2):e0297411. doi: 10.1371/journal.pone.0297411 (PMC10901330; doi:10.1371/journal.pone.0297411)
Supplement: Attachment — Submitted filename: Review_PONE-D-23-02018.pdf [file pone.0297411.s006.pdf]

PLOS ONE

Prevalence and determinants of contraceptive use among sexually active adolescents girls in 25 sub-Saharan African countries

--Manuscript Draft--

Manuscript Number:	PONE-D-23-02018
Article Type:	Research Article
Full Title:	Prevalence and determinants of contraceptive use among sexually active adolescents girls in 25 sub-Saharan African countries
Short Title:	Prevalence and determinants of contraceptive use among sexually active adolescents girls
Corresponding Author:	Turnwait Otu Michael, Ph.D.<br>University of Ibadan Faculty of Social Sciences<br>Ibadan, Oyo NIGERIA
Keywords:	Contraceptive use; demographic and health surveys; determinants; prevalence; sexually active adolescent girls; sub-Saharan Africa
Abstract:	Introduction<br><br>Various countries in sub-Saharan Africa have taken divergent steps toward achieving the Sustainable Development Goal's target of universal access to sexual and reproductive health-care services by 2030, particularly among sexually active adolescent girls who are at risk of unplanned pregnancies and sexually transmitted infections. However, due to a scarcity of multi-country empirical literature, we examined the prevalence and determinants of contraceptive use among adolescent girls who had been sexually active in the previous four weeks.<br><br>Materials and methods<br><br>We analyzed cross-sectional data from the most recent demographic and health surveys of 25 sub-Saharan African countries on 16,442 sexually active adolescent girls. In the analyses, descriptive statistics and multivariate binary logistic regression were used. Analyses were statistically significant at p<0.05.<br><br>Results<br><br>The overall prevalence of contraceptive use was 25.4%. Chad had the lowest prevalence (4%), while Namibia had the highest (60.5%). Over 90% of the countries studied had less than 50% contraceptive use among sexually active adolescent girls. Adolescent girls with at least secondary education were twice as likely as those with less than secondary education to use contraception (aOR = 1.58, 95% CI = 1.37-1.82). When compared to single adolescent girls, married adolescent girls were 66% less likely to use contraception (aOR = 0.34, 95% CI = 0.31-0.36). Adolescent girls with two or more children were seven times more likely than those without a child to use contraception (aOR = 6.91, 95% CI = 5.58-8.56).<br><br>Conclusion s<br><br>We found a low prevalence of contraceptive use among the girls in sub-Saharan Africa. As countries in the sub-region strive for universal access to reproductive health services, it is critical for the countries with low contraceptive use to include unrestricted free access to family planning services as a key component of policy interventions to meet the contraceptive needs of adolescent girls.
Order of Authors:	Turnwait Otu Michael, Ph.D.
	Tolulope Funmilola Ojo, M.Sc.
	Olasupo Augustine Ijabadeniyi, PhD
	Michael Ayodele Ibikunle, PhD

Powered by Editorial Manager® and ProduXion Manager® from Aries Systems Corporation

	James Olukayode Oni, M.Sc.
	Adebanke Adeorile Agboola, M.Sc.
**Additional Information:**	
**Question**	**Response**
**Financial Disclosure**<br><br>Enter a financial disclosure statement that describes the sources of funding for the work included in this submission. Review the submission guidelines for detailed requirements. View published research articles from *PLOS ONE* for specific examples.<br><br>This statement is required for submission and **will appear in the published article** if the submission is accepted. Please make sure it is accurate.<br><br>**Unfunded studies**<br>Enter: *The author(s) received no specific funding for this work.*<br><br>**Funded studies**<br>Enter a statement with the following details:<br>• Initials of the authors who received each award<br>• Grant numbers awarded to each author<br>• The full name of each funder<br>• URL of each funder website<br>• Did the sponsors or funders play any role in the study design, data collection and analysis, decision to publish, or preparation of the manuscript?<br>• **NO** - Include this sentence at the end of your statement: *The funders had no role in study design, data collection and analysis, decision to publish, or preparation of the manuscript.*<br>• **YES** - Specify the role(s) played.<br><br>* typeset	The author(s) received no specific funding for this work.
**Competing Interests**<br><br>Use the instructions below to enter a competing interest statement for this submission. On behalf of all authors, disclose any competing interests that could be perceived to bias this work—acknowledging all financial support and any other relevant financial or non-	The authors have declared that no competing interests exist.

financial competing interests.<br><br>This statement is required for submission and **will appear in the published article** if the submission is accepted. Please make sure it is accurate and that any funding sources listed in your Funding Information later in the submission form are also declared in your Financial Disclosure statement.<br><br>View published research articles from *PLOS ONE* for specific examples.<br><br>**NO authors have competing interests**<br><br>Enter: *The authors have declared that no competing interests exist.*<br><br>**Authors with competing interests**<br><br>Enter competing interest details beginning with this statement:<br><br>*I have read the journal's policy and the authors of this manuscript have the following competing interests: [insert competing interests here]*<br><br>* typeset	
**Ethics Statement**<br><br>Enter an ethics statement for this submission. This statement is required if the study involved:<br><br>• Human participants<br>• Human specimens or tissue<br>• Vertebrate animals or cephalopods<br>• Vertebrate embryos or tissues<br>• Field research<br><br>Write "N/A" if the submission does not require an ethics statement.<br><br>General guidance is provided below. Consult the submission guidelines for	N/A

Powered by Editorial Manager® and ProduXion Manager® from Aries Systems Corporation

detailed instructions. **Make sure that all information entered here is included in the Methods section of the manuscript.**<br><br>**Format for specific study types**<br><br>**Human Subject Research (involving human participants and/or tissue)**<br>• Give the name of the institutional review board or ethics committee that approved the study<br>• Include the approval number and/or a statement indicating approval of this research<br>• Indicate the form of consent obtained (written/oral) or the reason that consent was not obtained (e.g. the data were analyzed anonymously)<br><br>**Animal Research (involving vertebrate animals, embryos or tissues)**<br>• Provide the name of the Institutional Animal Care and Use Committee (IACUC) or other relevant ethics board that reviewed the study protocol, and indicate whether they approved this research or granted a formal waiver of ethical approval<br>• Include an approval number if one was obtained<br>• If the study involved *non-human primates*, add *additional details* about animal welfare and steps taken to ameliorate suffering<br>• If anesthesia, euthanasia, or any kind of animal sacrifice is part of the study, include briefly which substances and/or methods were applied<br><br>**Field Research**<br><br>Include the following details if this study involves the collection of plant, animal, or other materials from a natural setting:<br>• Field permit number<br>• Name of the institution or relevant body that granted permission	
**Data Availability**<br><br>Authors are required to make all data underlying the findings described fully available, without restriction, and from the time of publication. PLOS allows rare	Yes - all data are fully available without restriction

exceptions to address legal and ethical concerns. See the PLOS Data Policy and FAQ for detailed information.<br><br>A Data Availability Statement describing where the data can be found is required at submission. Your answers to this question constitute the Data Availability Statement and **will be published in the article**, if accepted.<br><br>**Important:** Stating 'data available on request from the author' is not sufficient. If your data are only available upon request, select 'No' for the first question and explain your exceptional situation in the text box.<br><br>Do the authors confirm that all data underlying the findings described in their manuscript are fully available without restriction?	
**Describe where the data may be found in full sentences. If you are copying our sample text, replace any instances of XXX with the appropriate details.**<br><br>• If the data are **held or will be held in a public repository**, include URLs, accession numbers or DOIs. If this information will only be available after acceptance, indicate this by ticking the box below. For example: *All XXX files are available from the XXX database (accession number(s) XXX, XXX.*).<br>• If the data are all contained **within the manuscript and/or Supporting Information files**, enter the following: *All relevant data are within the manuscript and its Supporting Information files.*<br>• If neither of these applies but you are able to provide **details of access elsewhere**, with or without limitations, please do so. For example:<br><br>*Data cannot be shared publicly because of [XXX]. Data are available from the XXX Institutional Data Access / Ethics Committee (contact via XXX) for researchers who meet the criteria for*	The data underlying the results presented in the study are available from DHS Program. The DHS datasets are publicly accessible at https://dhsprogram.com/data/.

Powered by Editorial Manager® and ProduXion Manager® from Aries Systems Corporation

access to confidential data.<br><br>*The data underlying the results presented in the study are available from (include the name of the third party and contact information or URL).*<br>• This text is appropriate if the data are owned by a third party and authors do not have permission to share the data.<br><br>* typeset	
Additional data availability information:	

Powered by Editorial Manager® and ProduXion Manager® from Aries Systems Corporation

Manuscript

Click here to access/download;Manuscript;Contraceptive use and adolescents Manuscript (PLOS ONE.docx

Prevalence and determinants of contraceptive use among sexually active adolescent girls in 25 sub-Saharan African countries

Turnwait Otu Michael[1]*, Tolulope Funmilola Ojo[2], Olasupo Augustine Ijabadeniyi[3], Michael Ayodele Ibikunle[3], James Olukayode Oni[4], Adebanke Adeorile Agboola[5]

[1] Department of Sociology, University of Ibadan, Ibadan, Nigeria

[2] Department of Public Health, Afe Babalola University, Ado-Ekiti, Nigeria

[3] Department of Sociology, Afe Babalola University, Ado-Ekiti, Nigeria

[4] Department of Pharmacology and Therapeutics, Afe Babalola University, Ado-Ekiti, Nigeria

[5] Department of Integrated Medical Sciences, Afe Babalola University, Ado-Ekiti, Nigeria

* Corresponding author

E-mail: turnwaitmichael@gmail.com (TOM)

Abstract

Introduction

Various countries in sub-Saharan Africa have taken divergent steps toward achieving the Sustainable Development Goal's target of universal access to sexual and reproductive health-care services by 2030, particularly among sexually active adolescent girls who are at risk of unplanned pregnancies and sexually transmitted infections. However, due to a scarcity of multi-country empirical literature, we examined the prevalence and determinants of contraceptive use among adolescent girls who had been sexually active in the previous four weeks.

Materials and methods

We analyzed cross-sectional data from the most recent demographic and health surveys of 25 sub-Saharan African countries on 16,442 sexually active adolescent girls. In the analyses, descriptive statistics and multivariate binary logistic regression were used. Analyses were statistically significant at $p<0.05$.

Results

The overall prevalence of contraceptive use was 25.4%. Chad had the lowest prevalence (4%), while Namibia had the highest (60.5%). Over 90% of the countries studied had less than 50% contraceptive use among sexually active adolescent girls. Adolescent girls with at least secondary education were twice as likely as those with less than secondary education to use contraception (aOR = 1.58, 95% Cl = 1.37-1.82). When compared to single adolescent girls, married adolescent girls were 66% less likely to use contraception (aOR = 0.34, 95% Cl = 0.31-0.36). Adolescent girls with two or more children were seven times more likely than those without a child to use contraception (aOR = 6.91, 95% Cl = 5.58-8.56).

Conclusions

We found a low prevalence of contraceptive use among the girls in sub-Saharan Africa. As countries in the sub-region strive for universal access to reproductive health services, it is critical for the countries with low contraceptive use to include unrestricted free access to family planning services as a key component of policy interventions to meet the contraceptive needs of adolescent girls.

Introduction

Adolescents are young people aged 10 to 19 [1]. In 2022, over 13 million births occurred among adolescents worldwide [2]. The Demographic and Health Survey collects information on the sexual and reproductive health (SRH) of adolescent girls starting at the age of 15 years [3]. Every year, approximately 21 million girls between the ages of 15 and 19 become pregnant in developing countries [1]. While 43 births per 1000 girls occurred worldwide in 2021, approximately 12 million of these births occurred in developing countries. Adolescent girls in Africa account for 14% of all babies born in Africa, compared to 9% globally [4]. While adolescent birth rates have declined in other parts of the world, sub-Saharan Africa (SSA) continues to have the highest (101 births per 1000 adolescent girls, exceeding the world average of 45 births per 1,000 adolescent girls), owing to a high unmet need for contraception among adolescents [4–6].

Contraception can be modern or traditional. Condoms, pills, implants, intra-uterine devices, and injectables are among the modern contraceptives that are more reliable and effective in preventing unwanted pregnancies [7,8]. Traditional contraceptives are unreliable and include withdrawal, periodic abstinence, herbal mixtures or concoctions [9,10]. Adolescents, like adults, are entitled to SRH rights, including access to counseling, contraception, and sexuality education, according to the World Health Organization [11].

Access to and use of modern contraception among sexually active adolescents has been shown to promote sexual behavior while also reducing maternal health risks, school dropout, and economic hardship in young girls [12]. Millions of adolescent girls, particularly in developing countries, lack access to contraception [13]. This has put many adolescent girls at risk of unplanned pregnancy, unsafe abortion, and maternal morbidity and mortality [11].

Though contraceptive use among adolescent girls varies by country, 14 million (43%) adolescent girls in low- and middle-income countries (LMICs) have an unmet need for modern contraception. Unintended pregnancies account for approximately 49% (10 million) of the 21 million pregnancies that occur among adolescents in LMICs each year; 50% of these pregnancies result in unsafe abortion [14]. Over 50% of adolescents in SSA have unmet contraception needs [15]. Babies born to adolescents who carried their pregnancies to term are 50% more likely to die, be born prematurely, or have serious health complications than babies born to older women [16–18].

Previous research had primarily focused on contraceptive use among adolescents in general, particularly at the country level [19–23]. At the multi-country level, no attention has been paid to sexually active adolescents' contraceptive use in SSA. A few multi-country level studies that examined contraceptive use among adolescents in SSA focused only on married/cohabited adolescents [24], young adult women aged 15-24 without disaggregation by adolescent age [25], and school-going adolescents (excluding out of school adolescents) [26]. The purpose of this study was to fill this void by assessing the prevalence and determinants of contraceptive use among sexually active adolescent girls in 25 SSA countries, in order to provide empirical evidence for advocacy and comparative policy options.

Materials and methods

Data source

The data for this study came from Demographic and Health Surveys (DHS) conducted in 25 SSA countries between January 1, 2012, and December 31, 2021. Table 1 shows the countries that were chosen. Individual recode files (women) were used. The most recent surveys from the countries chosen were used. The surveys from the included countries were selected because they all contain the key variables of interest for this study. The DHS collected information on adolescent girls and women's sexual activity, contraception, fertility, individual and household socioeconomic, and demographic factors. DHS is a five-year national survey conducted in LMICs. DHS employs a multi-stage sampling technique in all countries, beginning with cluster sampling of enumeration areas (EAs), followed by systematic selection of households, and simple random sampling of one illegible respondent from each household. This study sample (N = 16,442) consisted of sexually active adolescent aged 15-19 who responded to having been sexually active in the four weeks preceding the survey and had complete cases of variables of interest. Adolescents who stated that they had not been sexually active in the previous four weeks were excluded from the study. In writing this manuscript, the authors adhere to the 'Strengthening the Reporting of Observational Studies in Epidemiology' (STROBE) statement [27].

Table 1. Sample size and year of survey.

Country	Year	Weighted Sample	Weighted %
1) Angola	2015-16	961	5.8
2) Benin	2017-18	743	4.5
3) Burundi	2016-17	230	1.4
4) Chad	2014-15	1,164	7.1
5) Congo, DR*	2013-14	1,075	6.5
6) Ethiopia	2016	504	3.1

7) Gabon	2012	612	3.7
8) Gambia	2019-20	255	1.5
9) Ghana	2014	228	1.4
10) Guinea	2018	493	3.0
11) Kenya	2014	311	1.9
12) Lesotho	2014	182	1.1
13) Liberia	2019-20	498	3.0
14) Madagascar	2021	1,389	8.4
15) Malawi	2015-16	1,180	7.2
16) Mali	2018	789	4.8
17) Namibia	2013	248	1.5
18) Niger	2012	696	4.2
19) Nigeria	2018	1,913	11.6
20) Rwanda	2019-20	112	0.7
21) Sierra Leone	2019	972	5.9
22) South Africa	2016	241	1.5
23) Tanzania	2015-16	700	4.3
24) Zambia	2018	566	3.4
25) Zimbabwe	2015	384	2.3
**All Countries**	**-**	**16,442**	**100**

*Note: *DR = Democratic Republic; - = Not applicable*

Study variables

Outcome variable: The use of contraception by adolescent girls who were sexually active was the study's outcome variable. "Are you currently doing something or using any method to delay or avoid getting pregnant?" was the question posed to the respondents. "Which method are you using?" was the next question. Female and male sterilization, IUD, injectables, implants, pill, condom, female and male condoms, diaphragm, foam/jelly, lactational

amenorrhea, rhythm and withdrawal methods, and others were among the options presented to respondents. The responses as captured in the DHS datasets were categorized into ‘no method’, ‘folkloric method’, ‘traditional method’ and ‘modern method’. The folkloric and traditional methods were regrouped together as the traditional method for descriptive statistics for the purposes of this study. To create a binary outcome variable for logistic regression, the contraceptive method categories were recoded as follows: no method = 0, any method (traditional or modern) = 1.

Explanatory variables: Education, marital status, working status, ideal number of children, total number of children, ever had a terminated pregnancy, and knowledge of contraceptive method were the explanatory variables. Others were the number of sex partners, ability to request that a partner use a condom, ever been tested for HIV, wealth index, and type of residence, as well as heard family planning on radio, TV, newspaper/magazine in the last few months. Education was originally coded as no education = 0, primary education = 1, secondary education = 2, and higher education = 3. This was recoded as follows: below secondary = 1 and secondary+ = 2. The marital status was recoded as follows: never married = 0, ever married = 1. Working status was coded as 0 (not working) or 1 (working). The ideal number of children was recoded as 0-2 = 1, 3-5 = 2, and 6 or more = 3. The total number of children born was recoded as none = 0, one = 1, two or more = 2. Never had a termination of pregnancy was coded as No = 0, Yes = 1. Contraceptive method knowledge was recoded as follows: knows no method = 0, knows any method (including folkloric, traditional, and modern methods) = 2. Family planning overheard on the radio was coded (No = 0, Yes = 1). On the television, family planning heard was coded as No = 0, Yes = 1. In the newspaper/magazine, family planning heard was coded as No = 0, Yes = 1. The number of sexual partners was recoded as one = 1, two or more = 2. The ability to request that a partner use a condom was coded as No = 0 and Yes = 1. Ever tested for HIV was coded as No = 0 and Yes = 1. Poorest

= 1, Poorer = 2, Middle = 3, Richer = 4, and Richest = 5 were assigned to the wealth index. The location of residence was coded as Urban = 1, Rural = 2. The DHS dataset's wealth index was computed in quintiles using Principal Component Analysis (PCA), capturing respondents' ownership of household assets such as bicycles, televisions, water sources, toilet facilities, and building materials[28]. The availability of the explanatory variables in the DHS datasets influenced their selection. A previous review of the literature on the relationship between determinants of contraceptive use also supported variable selection [3,18,20].

Statistical analyses

The statistical analysis was carried out in three steps. The first was the use of univariate analysis to describe sample and explanatory variables. The second was the use of bivariate analysis on explanatory variables against contraceptive use by methods (none, traditional, or modern) via crosstabulation and chi-square statistics. The chi-square test was accepted at $p<0.05$. Only variables that were significant in the chi-square test advanced to the final stage. Variance inflation factor (VIF) was used for collinearity diagnostics to check for multicollinearity among predictor variables. According to the diagnostic test results, none of the explanatory variables had collinearity. Minimum VIF = 1.02 and maximum VIF = 1.40. The reference groups were chosen based on normative categories, the lowest likelihood of using any contraception method as documented in the literature, and/or the largest category of observations. To avoid oversampling and under-sampling errors, the DHS datasets were weighted using the women's unit of analysis (v005) at "COMPUTE WGT = V005/1000000. WEIGHT by WGT." To avoid confounding effects of explanatory variables, non-sexually active adolescent girls, as well as missing and non-responses, were dropped from the analysis. SPSS Statistics 25 software was used for the analysis, and all statistics were tested at a confidence level of 0.95.

Ethical clearance

The authors obtained permission from the DHS Program to use DHS datasets in this study. The DHS received ethical approval from the ICF Institutional Review Board (ICF IRB

FWA00000845) in the United States, as well as from the National Health Research Ethics Committees of the countries chosen for this study. In Nigeria, for example, the National Health Research Ethics Committee of Nigeria granted ethical approval for the survey (NHREC/01/01/2007). The DHS datasets are publicly accessible at https://dhsprogram.com/data/.

Results

Fig 1 depicts the prevalence of contraceptive use among sexually active adolescent girls in SSA. According to the findings, the overall prevalence of contraceptive use in SSA is 25.4%, with rates ranging from 4% in Chad to 60.5% in Namibia. Chad (4%), Nigeria (5.4%), Niger (7.2%), and Gambia (8.2%) have lower than 10% contraceptive use. Lesotho (53.6%), South Africa (59.8%), and Namibia (60.5%) have more than 50% contraceptive use.

Fig 1. Prevalence (percentage) of modern contraceptive use among sexually active adolescent girls in SSA.

Table 2 presents the results regarding background characteristics and contraceptive use among sexually active adolescent girls in SSA. Sixty-four percent of adolescent girls had less than a secondary education, 66.7% were married, 43.7% were working, 43.4% had at least one child, 6.9% had terminated a pregnancy in their lifetime, 90.3% knew at least one contraceptive method, and 25.9%, 11.6%, and 5.0% had heard about family planning on radio, television, and newspaper/magazine, respectively. In the previous 12 months, 10% of respondents had two or more sex partners, 33.9% had been tested for HIV, 22.3% came from the poorest households, and 69.3% lived in rural areas. The chi-square statistical tests revealed that all of the independent variables were significantly associated with adolescent girl contraceptive use.

Table 2. Background characteristics and contraceptive use among sexually active adolescent girls in SSA.

Variables	Weighted (N = 16,442)		Contraceptive Method Type			
	n	%	None (%)	Traditional (%)	Modern (%)	p-values
**Education**						<0.001
Below Secondary	10,475	63.7	79.9	1.9	18.6	
Secondary+	5,966	36.3	57.2	5.5	37.3	
**Marital status**						<0.001
Never in union	5,469	33.3	56.3	5.3	38.4	
Ever married	10,973	66.7	79.3	1.8	18.9	
**Working status**						0.947
Not working	9,235	56.3	71.7	2.9	25.4	
Working	7,178	43.7	71.5	3.0	25.5	
**Ideal number of children**						<0.001
0-2	1,998	12.7	56.9	3.0	40.1	
3-5	8,764	55.6	64.3	3.7	32.0	
6+	5,013	31.8	87.8	1.9	10.3	
**Total children ever born**						<0.001
None	9,307	56.6	75.5	3.3	21.2	
1	5,747	35.0	64.3	2.6	33.1	
2 or more	1,388	8.4	76.3	2.2	21.5	
**Ever had a terminated pregnancy**						<0.001
No	15,295	93.1	71.3	2.9	25.9	
Yes	1,141	6.9	77.0	4.1	18.9	
**Knowledge of contraceptive method**						<0.001
Knows no method	1,598	9.7	100.0	0.0	0.0	
Knows any method	14,845	90.3	68.6	3.3	28.1	
**Heard family planning on radio last few months**						<0.001
No	12,177	74.1	74.0	2.8	23.2	

Yes	4,258	25.9	65.0	3.5	31.5	
**Heard family planning on TV last few months**						<0.001
No	14,534	88.4	73.2	2.7	24.1	
Yes	1,904	11.6	60.3	5.1	34.6	
**Heard family planning in newspaper/magazine last few months**						<0.001
No	15,628	95.0	72.6	2.9	24.6	
Yes	814	5.0	54.7	4.5	40.8	
**Number of sex partners in last 12 months**						<0.001
1	14,819	90.1	72.5	2.7	24.8	
2 or more	1622	9.9	64.1	5.1	30.8	
**Respondents can ask partner to use condom**						<0.001
No	4,558	49.0	86.7	1.2	12.0	
Yes	4,183	44.9	68.0	2.5	29.5	
Don't know	565	6.1	87.1	1.4	11.5	
**Ever been tested for HIV**						
No	9134	66.1	75.0	3.5	21.5	<0.001
Yes	4679	33.9	55.7	2.4	41.9	
**Wealth index**						<0.001
Poorest	3,665	22.3	78.7	2.0	19.3	
Poorer	3,789	23.0	75.5	2.1	22.5	
Middle	3,560	21.6	71.4	3.3	25.3	
Richer	3,137	19.1	67.3	3.6	29.2	
Richest	2,291	13.9	60.6	4.6	34.8	
**Type of residence**						<0.001
Urban	5,042	30.7	63.1	4.4	32.5	
Rural	11,400	69.3	75.5	2.3	22.2	

The unadjusted and adjusted odds ratios of binary logistic regression of contraceptive use among adolescent girls in SSA are shown in Table 3. Education, marital status, ideal number of children, number of children ever born, heard family planning on radio, ability to request that a partner use a condom, ever been tested for HIV, wealth index, and residence were all significantly associated with contraceptive use among adolescent girls in the unadjusted logistic regression model. In the adjusted model, adolescent girls with at least secondary education were twice as likely as those with less than secondary education to use contraception. When compared to single adolescent girls, married adolescent girls were 66% less likely to use contraception. Adolescent girls with an ideal number of 6 or more children were 70% less likely to use contraception than those with an ideal number of 0-2 children. Adolescent girls with two or more children were seven times more likely than those without a child to use contraception. Adolescent girls who heard about family planning on the radio [aOR = 1.25, 95% Cl = 1.09-1.44] or in the newspaper/magazine [aOR = 1.43, 95% Cl = 1.04-1.96] were more likely to use contraception than those who did not. Adolescent girls who could request that their partners use a condom were twice as likely as those who couldn't. Girls who had ever been tested for HIV were twice as likely as those who had never been tested to use contraception. Others were more likely to use contraception than adolescent girls from the poorest households. Surprisingly, adolescent girls in rural areas were twice as likely as those in urban areas to use contraception (both modern and traditional methods combined). However, as shown in Table 2, adolescent girls in urban areas (32.5%) were more likely to use modern contraception than those in rural areas (22.2%).

Table 3. Binary logistic regression of contraceptive use among adolescent girls in SSA.

Variables	Use no method n (%)	Use any method n (%)	Unadjusted Odds Ratio (95% CI)	Adjusted Odds Ratio (95% CI)
**Education**				
Below Secondary	8370 (79.9)	2105 (20.1)	Ref	Ref
Secondary+	3413 (57.2)	2554 (42.8)	1.64 (1.42-1.89)***	1.58 (1.37-1.82)***
**Marital status**				
Never in union	3079 (56.3)	2390 (43.7)	Ref	Ref
Ever married	8705 (79.3)	2268 (20.7)	0.34 (0.32-0.37)***	0.34 (0.31-0.36)***
**Ideal number of children**				
0-2	1137 (56.9)	860 (43.1)	Ref	Ref
3-5	5635 (64.3)	3130 (35.7)	0.80 (0.68-0.96)***	0.83 (0.69-0.98)*
6+	4402 (87.8)	611 (12.2)	0.32 (0.26-0.39)***	0.30 (0.24-0.37)***
**Total children ever born**				
None	7029 (75.5)	2278 (24.5)	Ref	Ref
1	3696 (64.3)	2052 (35.7)	8.58 (7.31-10.06)***	8.41 (7.19-9.83)***
2 or more	1059 (76.4)	328 (23.6)	7.37 (5.97-9.11)***	6.91 (5.58-8.56)***
**Ever had a terminated pregnancy**				
No	10900 (71.3)	4395 (28.7)	Ref	Ref
Yes	878 (77.0)	263 (23.0)	0.96 (0.76-1.20)	0.89 (0.71-1.13)
**Heard family planning on radio last few months**				
No	9013 (74.0)	3164 (26.0)	Ref	Ref
Yes	2768 (65.0)	1490 (35.0)	1.37 (1.18-1.58)***	1.25 (1.09-1.44)**
**Heard family planning on TV last few months**				
No	10636 (73.2)	3897 (26.8)	Ref	Ref
Yes	1147 (60.2)	757 (39.8)	0.82 (0.65-1.012)	0.80 (0.65-0.99)
**Heard family planning in newspaper/magazine last few months**				
No	11339 (72.6)	4289 (27.4)	Ref	Ref

Yes	445 (54.7)	369 (45.3)	1.21 (0.87-1.68)	1.43 (1.04-1.96)*
**Respondents can ask partner to use condom**				
No/Don't know	4446 (86.8)	678 (13.2)	Ref	Ref
Yes	2844 (68.0)	1338 (32.0)	1.78 (1.56-2.02)***	1.71 (1.50-1.94)***
**Ever been tested for HIV**				
No	6852 (75.0)	2282 (25.0)	Ref	Ref
Yes	2605 (55.7)	2073 (44.3)	2.21 (1.94-2.51)***	2.12 (1.86-2.41)***
**Wealth index**				
Poorest	2886 (78.7)	779 (21.3)	Ref	Ref
Poorer	2860 (75.5)	929 (24.5)	1.18 (0.99-1.40)***	1.19 (1.00-1.42)*
Middle	2540 (71.4)	1019 (28.6)	1.21 (1.01-1.45)*	1.22 (1.02-1.47)*
Richer	2110 (67.2)	1028 (32.8)	1.34 (1.09-1.64)**	1.36 (1.11-1.66)**
Richest	1388 (60.6)	903 (39.4)	1.89 (1.48-2.43)***	1.23 (1.73-2.88)***
**Type of residence**				
Urban	3181 (63.1)	1861 (36.9)	Ref	Ref
Rural	8603 (75.5)	2797 (24.5)	1.40 (1.18-1.65)***	1.53 (1.29-1.81)***
Model chi-square			1959.819***	1915.452***
-2 Log Likelihood			6508.994	6503.075
Cox & Snell R^2			0.231	0.231
Nagelkerke R^2			0.340	0.337
Classification overall % (correct)			79.7	78.8
N	16,442	16,442	16,442	16,442

Significant at p < .05, p < .01**, p < .001***; Ref – reference category; CI – confidence interval;* n *– weighted count; % - weighted percentage*

Discussion

We investigated the prevalence of contraceptive use among adolescent girls who were sexually active in 25 SSA countries. The overall prevalence of contraceptive use was 25.4%. Chad had the lowest prevalence (4%), while Namibia had the highest (60.5%). Contraception use is lower

than 10% in Chad (4%), Nigeria (5.4%), Niger (7.2%), and Gambia (8.2%). Lesotho (53.6%), South Africa (59.8%), and Namibia (60.5%) have more than 50% contraceptive use. The fact that 23 out of 25 SSA countries studied, or 92% of the countries sampled, had less than 50% contraceptive use among adolescent girls who were sexually active confirms the assertion that adolescents in SSA continue to face significant sexual and reproductive health challenges [26]. Similarly, a previous study of adolescent girls in 32 SSA countries that did not specifically target sexually active adolescent girls found an overall low prevalence of contraceptive use of 18.9%. [29]. Another study conducted in 26 SSA found 22.6% low prevalence of contraceptive use among adolescent girls in general, with no specificity for sexually active adolescent girls [30]. Low contraceptive use among girls and women in SSA, particularly in Chad, is linked to socio-cultural and religious norms that prevent girls from accessing reproductive health services [31].

In our study, sexually active adolescent girls with at least secondary education were more likely to use contraception than those with less than secondary education. Previous research in SSA found a link between educational level and contraceptive use among adolescents [18,20,32]. Education boosts adolescents' self-esteem, confidence, reproductive health rights, and willingness to use contraception to avoid unwanted pregnancy [22,23]. We found that married adolescent girls were less likely to use contraception than unmarried adolescent girls. Adolescent girls in SSA face the challenge of early child marriage, which is promoted by some cultural practices that emphasize virginity and large family size; as a result, married adolescents are less likely to use contraception [10,33]. Contraception is thought to encourage promiscuity in adolescent girls, which discourages husbands from allowing their younger wives to use contraception [32,34].

We found that having a larger ideal number of children was associated with less contraceptive use. Adolescent girls who had an ideal number of 6 or more children, for

example, were less likely to use contraception than those who had an ideal number of 0-2 children. Previous studies in SSA found that cultural beliefs and a desire for a large family size discourage married girls from using contraception [24,35]. A similar study in SSA discovered that adolescent girls with ideal family sizes of six or more children were less likely to use contraception [24]. Our study found that adolescent girls with two or more children were more likely to use contraception than those without a child. According to studies, the desire to avoid another childbearing experience or an unplanned pregnancy prompted girls to use contraception [36,37]. Furthermore, counseling obtained during antenatal and postnatal examinations has provided some adolescent mothers with knowledge and access to family planning in order to avoid unplanned pregnancy [38,39].

We found that adolescent girls who heard about family planning on the radio or in the newspaper/magazine were more likely than those who did not to use contraception. Previous studies have shown that media exposure to family planning, whether through radio or newspaper reading, improves adolescents' contraceptive use [21,40,41]. Radio is an important media source for disseminating family planning information to adolescent girls in rural areas and to groups of young people who are not literate enough to read and understand, thanks to local radio stations that broadcast in indigenous languages [42,43]. Newspapers and magazines are also important communication channels for educating the young population about family planning [21]. The texts and diagrams used to convey messages aid in the retention of family planning information, particularly about various methods of contraception, in the minds of the readers.

According to our findings, adolescent girls who could request that their partners use a condom were more likely to use contraception than those who couldn't. The ability to request that a partner use a condom indicates that a girl has autonomy and control over her SRH issues. According to studies, female decision-making power over SRH promotes sexual equality and

helps to prevent unplanned pregnancy [29,40]. We found that girls who had ever been tested for HIV were more likely to use contraception than those who had never been tested. This could be because a girl seeking HIV testing has the opportunity to receive counseling and information about the importance of abstinence or the use of condoms to prevent sexually transmitted infections. Using a condom to prevent HIV and other sexually transmitted infections encourages the use of contraception [10].

Adolescent girls from other households were found to be more likely to use contraception than adolescent girls from the poorest households. Adolescent girls from wealthier households use contraception more frequently, because they have enough money to cover the cost of contraception. Previous research found that female adolescents in the richest wealth quintile were more likely to use contraception than female adolescents in the poorest wealth quintile [21,29,41]. When we combined modern and traditional methods, we discovered that adolescent girls in rural areas were more likely than those in urban areas to use any contraception method. Adolescent girls in urban areas, on the other hand, were more likely to use modern contraception than those in rural areas. Several other studies in SSA found that adolescents in urban areas use modern contraception more than their counterparts in rural areas [10,22,24].

Strengths and limitations

The study's strength is that it uses standardized and validated nationally representative datasets from 25 SSA countries, which improves the generalizability of the study's findings to many African societies. Furthermore, the study used the most recent DHS datasets from the selected SSA countries, so our findings reflect current realities on the continent. Nonetheless, the study has some limitations. First, the data used in the study came from cross-sectional surveys, which gathered information from respondents at a specific point in time, making longitudinal behavioral changes among adolescent girls difficult to obtain. In addition, because the data

used were from cross-sectional surveys, our analytical findings were prevented from presenting a causal-effect relationship, which is common in experimental research. More so, generalizing our study findings across the entire SSA should be done with caution because the study only covered 25 countries in SSA and did not include the entire region. Furthermore, because the study used the most recent DHS datasets from the selected countries, there were tendencies of variations in years of surveys, which could influence findings, particularly on prevalence of contraceptive use across countries in SSA, as recent surveys due to modernization and policy improvement in some countries may favor more contraceptive use among young people. Finally, because the surveys were conducted on human subjects and some interview questions were retrospective in nature, the possibility of forgetfulness should not be completely ruled out in the interpretation and generalization of our study findings.

Conclusions

In general, we found a low prevalence of contraceptive use among sexually active adolescent girls in SSA. As SSA countries strive to achieve the SDGs' target of universal access to SRH services, including family planning, and reducing unmet need for modern contraception, particularly among adolescent girls, it is critical for SSA countries with low contraceptive use to include unrestricted free access to family planning services as part of policy interventions to meet the contraceptive demands of its teeming young population. Both social media and mainstream media should be used to raise awareness about contraceptive use, using English, pidgin, and indigenous languages as communication tools to reach out to all types of young girls, including educated and uneducated, rich and poor, rural and urban dwellers. Efforts must also be made to reduce child marriage in the SSA region, as adolescent girls who marry earlier miss out on formal education and are exposed to risky early motherhood due to a lack of empowerment for SRH rights.

// Acknowledgments
We are grateful to Measure DHS for granting us permission to use DHS datasets in this study.

Author Contributions

Conceptualization: Turnwait Otu Michael, Tolulope Funmilola Ojo, Olasupo Augustine Ijabadeniyi, Michael Ayodele Ibikunle, James Olukayode Oni, Adebanke Adeorile Agboola.

Data curation: Turnwait Otu Michael.

Formal analysis: Turnwait Otu Michael.

Methodology: Turnwait Otu Michael, Tolulope Funmilola Ojo.

Supervision: Turnwait Otu Michael, Tolulope Funmilola Ojo, Olasupo Augustine Ijabadeniyi, Michael Ayodele Ibikunle, James Olukayode Oni, Adebanke Adeorile Agboola.

Visualization: Turnwait Otu Michael, Tolulope Funmilola Ojo, Olasupo Augustine Ijabadeniyi, Michael Ayodele Ibikunle, James Olukayode Oni, Adebanke Adeorile Agboola.

Writing – original draft: Turnwait Otu Michael, Tolulope Funmilola Ojo, Olasupo Augustine Ijabadeniyi, Michael Ayodele Ibikunle, James Olukayode Oni.

Writing – review & editing: Turnwait Otu Michael, Tolulope Funmilola Ojo, Olasupo Augustine Ijabadeniyi, Michael Ayodele Ibikunle, James Olukayode Oni, Adebanke Adeorile Agboola.

References

1. World Health Organization (WHO). Adolescent pregnancy. 15 Sep 2022 [cited 21 Jan 2023]. Available: https://www.who.int/news-room/fact-sheets/detail/adolescent-pregnancy
2. World Population Review. Teenage Pregnancy Rates by Country 2022. In: World Population Review [Internet]. 2023 [cited 21 Jan 2023]. Available: https://worldpopulationreview.com/country-rankings/teenage-pregnancy-rates-by-country

3. National Population Commission (NPC) [Nigeria] and ICF. Nigeria Demographic and Health Survey 2018. Abuja, Nigeria; 2019. Available: http://nationalpopulation.gov.ng/ndhs-2018-key-finding/

4. You D, Hug L, Anthony D. UNICEF report Generation 2030 Africa calls upon investing in and empowering girls and young women. Reprod Health. 2015;12: 1–4. doi:10.1186/S12978-015-0007-X/FIGURES/3

5. United Nations D of E and SA. World Population Prospects - Population Division - United Nations. In: United Nations [Internet]. 2022 [cited 21 Jan 2023]. Available: https://population.un.org/wpp/Download/Standard/Fertility/

6. United Nations Children's Fund (UNICEF). Generation 2030 Africa 2.0: Prioritizing investments in children to reap the demographic dividend. 2nd ed. New York: United Nations Children's Fund (UNICEF) Division of Data, Research and Policy; 2017. Available: www.unicef.org/publications/index_101219.htmlhttps://au.int/web/sites/default/files/pages/31829-file-african-union-handbook-2017-edited.pdf.

7. Kantorová V, Wheldon MC, Ueffing P, Dasgupta ANZ. Estimating progress towards meeting women's contraceptive needs in 185 countries: A Bayesian hierarchical modelling study. PLoS Med. 2020;17. doi:10.1371/JOURNAL.PMED.1003026

8. National Bureau of Statistics (NBS), United Nations Children's Fund (UNICEF). Multiple Indicator Cluster Survey 2021, Survey Findings Report. Abuja, Nigeria; 2022 Aug. Available: https://www.unicef.org/nigeria/media/6316/file/2021%20MICS%20full%20report%20.pdf

9. Boglaeva L v. Contraceptive method mix in the context of family planning programmes in developing countries. Popul Econ. 2021;5: 56–75. doi:10.3897/POPECON.5.E70669

10. Agbana RD, Michael TO, Ojo TF. Family planning method discontinuation among Nigerian women: Evidence from the Nigeria Demographic and Health Survey 2018. J Taibah Univ Med Sci. 2023;18: 117–124. doi:10.1016/j.jtumed.2022.08.003

11. World Health Organization (WHO). Sexual and Reproductive Health and Research (SRH). In: World Health Organization [Internet]. 2022 [cited 21 Jan 2023]. Available: https://www.who.int/teams/sexual-and-reproductive-health-and-research-(srh)/areas-of-work/adolescent-and-sexual-and-reproductive-health-and-rights

12. Michael TO, Nwokocha EE, Ukwandu D. CHILD DELIVERY CARE PRACTICES AMONG UNMARRIED YOUNGER ADOLESCENTS IN NIGERIA: The Case of Akwa Ibom State. Nigerian Journal of Economic and Social Studies. 2021;63: 403–432. Available: https://www.njess.org/journal/njess/articles?id=70

13. World Health Organization (WHO). Adolescent pregnancy. World Health Organization. 2019. Available: https://apps.who.int/iris/bitstream/handle/10665/329883/WHO-RHR-19.15-eng.pdf

14. Sully E, Biddlecom A, Darroch J, Riley T, Ashford L, Lince-Deroche N, et al. Adding It Up: Investing in Sexual and Reproductive Health 2019. New York; 2020. Available: https://www.guttmacher.org/sites/default/files/report_pdf/adding-it-up-investing-in-sexual-reproductive-health-2019.pdf

15. United Nations D of E and SA. Adolescent Fertility since the International Conference on Population and Development (ICPD) in Cairo. 2013. Available: https://www.un.org/en/development/desa/population/publications/pdf/fertility/Report_Adolescent-Fertility-since-ICPD.pdf

16. Ganchimeg T, Ota E, Morisaki N, Laopaiboon M, Lumbiganon P. Pregnancy and childbirth outcomes among adolescent mothers: a World Health Organization multicountry study. Wiley Online Library. 2014;121 Suppl 1: 40–48. doi:10.1111/1471-0528.12630

17. Darroch J, Woog V, Bankole A, Ashford L. Adding it up: Costs and Benefits of Meeting the Contraceptive Needs of Adolescents | Guttmacher Institute. 2016 May. Available: https://www.guttmacher.org/report/adding-it-meeting-contraceptive-needs-of-adolescents

18. Chola M, Hlongwana K, Ginindza TG. Patterns, trends, and factors associated with contraceptive use among adolescent girls in Zambia (1996 to 2014): a multilevel analysis. BMC Womens Health. 2020;20. doi:10.1186/S12905-020-01050-1

19. Nsanya MK, Atchison CJ, Bottomley C, Doyle AM, Kapiga SH. Modern contraceptive use among sexually active women aged 15–19 years in North-Western Tanzania: results from the Adolescent 360 (A360) baseline survey. BMJ Open. 2019;9: e030485. doi:10.1136/BMJOPEN-2019-030485

20. Casey SE, Gallagher MC, Kakesa J, Kalyanpur A, Muselemu JB, Rafanoharana RV, et al. Contraceptive use among adolescent and young women in North and South Kivu, Democratic Republic of the Congo: A cross-sectional population-based survey. PLoS Med. 2020;17. doi:10.1371/JOURNAL.PMED.1003086

21. Sserwanja Q, Turimumahoro P, Nuwabaine L, Kamara K, Musaba MW. Association between exposure to family planning messages on different mass media channels and the utilization of modern contraceptives among young women in Sierra Leone: insights from the 2019 Sierra Leone Demographic Health Survey. BMC Womens Health. 2022;22. doi:10.1186/S12905-022-01974-W

22. Ahissou NCA, Benova L, Delvaux T, Gryseels C, Dossou J-P, Goufodji S, et al. Modern contraceptive use among adolescent girls and young women in Benin: a mixed-methods study. BMJ Open. 2022;12: e054188. doi:10.1136/bmjopen-2021-054188

23. Pleaner M, Kutywayo A, Beksinska M, Mabetha K, Naidoo N, Mullick S. Knowledge, uptake and patterns of contraception use among in-school adolescents in three South African townships: Baseline findings from the Girls Achieve Power (GAP Year) Trial. Gates Open Res. 2022;6: 67. doi:10.12688/gatesopenres.13636.1

24. Ahinkorah BO. Predictors of modern contraceptive use among adolescent girls and young women in sub-Saharan Africa: a mixed effects multilevel analysis of data from 29 demographic and health surveys. Contracept Reprod Med. 2020;5. doi:10.1186/S40834-020-00138-1

25. Behrman JA, Wright KQ, Grant MJ, Soler-Hampejsek E. Trends in Modern Contraceptive Use among Young Adult Women in sub-Saharan Africa 1990 to 2014. Stud Fam Plann. 2018;49: 319–344. doi:10.1111/SIFP.12075

26. James PB, Osborne A, Babawo LS, Bah AJ, Margao EK. The use of condoms and other birth control methods among sexually active school-going adolescents in nine sub-Saharan African countries. BMC Public Health. 2022;22. doi:10.1186/S12889-022-14855-6

27. Vandenbroucke JP, von Elm E, Altman DG, Gøtzsche PC, Mulrow CD, Pocock SJ, et al. Strengthening the Reporting of Observational Studies in Epidemiology (STROBE): explanation and elaboration. Int J Surg. 2014;12: 1500–1524. doi:10.1016/J.IJSU.2014.07.014

28. Rutstein SO, Johnson K. The DHS wealth index. Calverton, Maryland, USA; 2004 Aug. Available: https://dhsprogram.com/publications/publication-cr6-comparative-reports.cfm

29. Ahinkorah BO, Hagan JE, Seidu AA, Sambah F, Adoboi F, Schack T, et al. Female adolescents' reproductive health decision-making capacity and contraceptive use in sub-Saharan Africa: What does the future hold? PLoS One. 2020;15. doi:10.1371/JOURNAL.PONE.0235601

30. Dwomoh D, Amuasi S, Amoah E, Gborgbortsi W, Tetteh J. Exposure to family planning messages and contraceptive use among women of reproductive age in sub-Saharan Africa: a cross-sectional program impact. Sci Rep. 2022;12: 18941. Available: https://www.nature.com/articles/s41598-022-22525-1

31. Adde KS, Ameyaw EK, Mottey BE, Akpeke M, Amoah RM, Sulemana N, et al. Health decision-making capacity and modern contraceptive utilization among sexually active women: Evidence from the 2014–2015 Chad Demographic and Health Survey. Contracept Reprod Med. 2022;7. doi:10.1186/S40834-022-00188-7

32. Ehiaghe A, Barrow A. Parental knowledge, willingness, and attitude towards contraceptive usage among their unmarried adolescents in Ekpoma, Edo State, Nigeria. Int J Reprod Med. 2022; 1–13. Available: https://www.hindawi.com/journals/ijrmed/2022/8533174/

33. Olamijuwon E, Odimegwu C. Saving Sex for Marriage: An Analysis of Lay Attitudes Towards Virginity and its Perceived Benefit for Marriage. Sex Cult. 2022;26: 568–594. doi:10.1007/S12119-021-09909-7

34. Michael T, Nwokocha E, Agbana R. Issues in Antenatal Care Services Utilization Among Unmarried Adolescents in Akwa Ibom State, Nigeria. Journal of Population and Social Studies [JPSS]. 2023;31: 359–380. doi:10.25133/JPSSv312023.021

35. Michael TO, Scent G. Correlates of Contraceptive Use and the Desire for less Children in Nigeria. The Nigerian Journal of Sociology and Anthropology. 2017;15: 101–116. doi:10.36108/NJSA/7102/51(0270)

36. Hall J, Barrett G, Rocca C. Evaluation of the Desire to Avoid Pregnancy Scale in the UK: a psychometric analysis including predictive validity. BMJ Open. 2022;12. Available: https://bmjopen.bmj.com/content/12/7/e060287.abstract

37. Chace Dwyer S, Baruwa S, Okafor E, Daini BO, Ubuane O, Jain A. How do changes in motivation to prevent pregnancy influence contraceptive continuation? Results from a longitudinal study with women who receive family planning services from Community Pharmacists and Patent and Proprietary Medicine Vendors in Nigeria. Reprod Health. 2022;19. doi:10.1186/S12978-022-01326-9

38. Barbieri MM, Dantas-Silva A, Caleffi LS, Morais SS, Juliato CRT, Bahamondes L, et al. One-year follow-up of immediate postpartum contraceptive implant insertion in adolescents. European

Journal of Contraception and Reproductive Health Care. 2022; 1–7. doi:10.1080/13625187.2022.2133536

39. Roque C, Morello L, Arora K. Postpartum Contraceptive Decision-Making of Parous Teens–A Qualitative Study. J Pediatr Adolesc Gynecol. 2022;35: 329–335. Available: https://www.sciencedirect.com/science/article/pii/S1083318821003314

40. Appiah F, Seidu A, Ahinkorah B, Baatiema L, Ameyaw E. Trends and determinants of contraceptive use among female adolescents in Ghana: analysis of 2003–2014 demographic and health surveys. SSM - Population Health . 2020;10: 100554. Available: https://www.sciencedirect.com/science/article/pii/S2352827319303805

41. Sharma H, Singh SK. Socioeconomic inequalities in contraceptive use among female adolescents in south Asian countries: a decomposition analysis. BMC Womens Health. 2022;22. doi:10.1186/S12905-022-01736-8

42. Boamah-Kaali EA, C Ruiter RA, Rodriguez MJ, Enuameh Y, Owusu-Agyei S, Poku Asante K, et al. "Family Planning Is Not a Bad Thing": A Qualitative Study of Individual Level Factors Explaining Hormonal Contraceptive Uptake and Consistent Use Among. Taylor & Francis. 2022; 1–21. doi:10.1080/23293691.2022.2140618

43. Browne L, Cooper S, Tiendrebeogo C, Bicaba F, Bila A, Bicaba A, et al. Using experience to create evidence: a mixed methods process evaluation of the new free family planning policy in Burkina Faso. Reprod Health. 2022;19. doi:10.1186/S12978-022-01375-0

Fig 1.

Click here to access/download;Figure;Fig1.tif

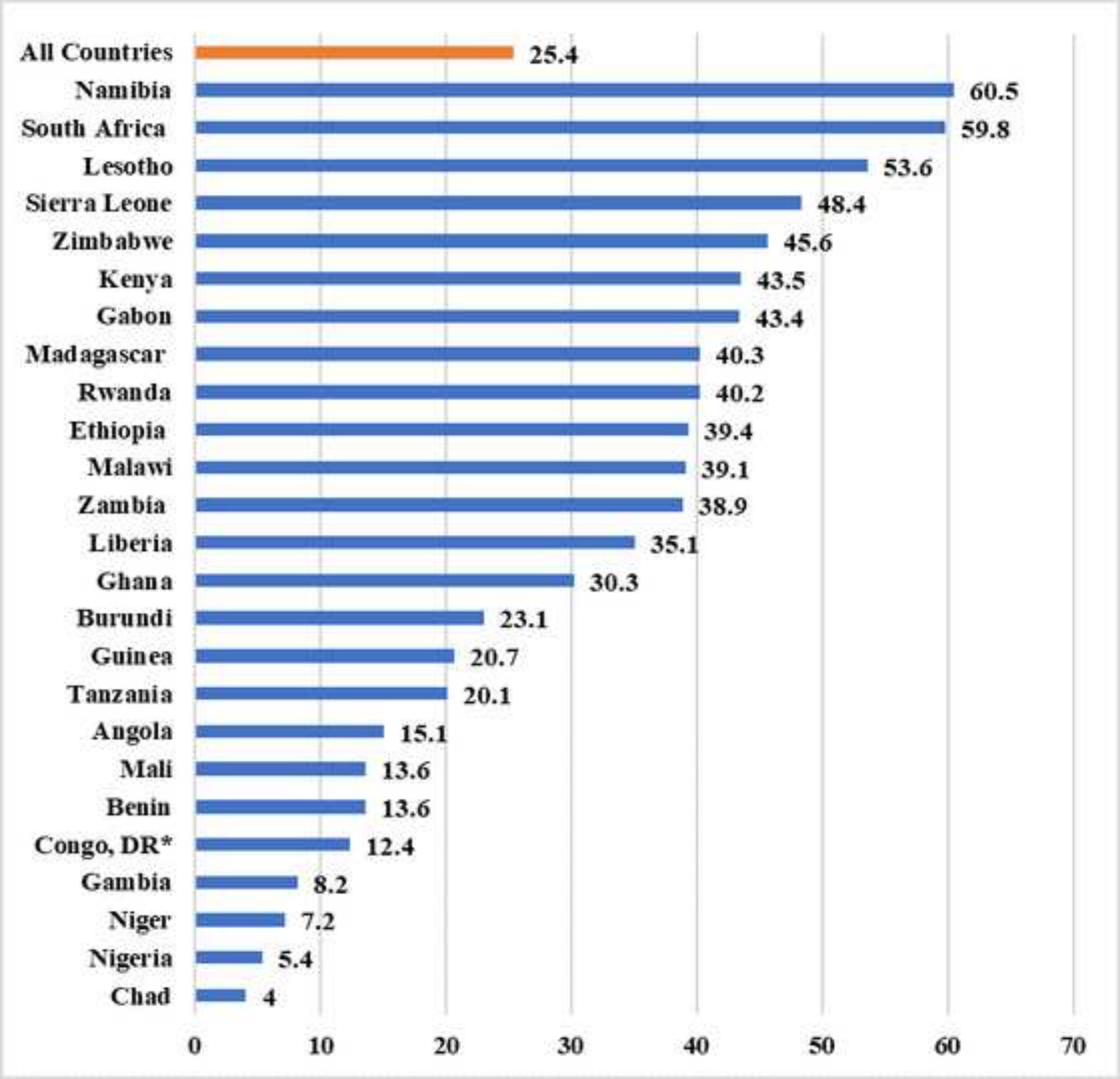

Click here to access/download
Supporting Information
S1 Appendix.doc

Click here to access/download
Supporting Information
S2 VIF Collinearity Statistics Results.docx

Logistic Regression Unadjusted Odds Ratio Result

Click here to access/download

Supporting Information

S3 Logistic Regression - Unadjusted Odds Ratio Result.docx

Logistic Regression Adjusted Odds Ratio Result

Click here to access/download
Supporting Information
S4 Logistic Regression - Adjusted Odds Ratio Result.docx

Click here to access/download
Supporting Information
S5 DHS Authorization Letter.pdf

Data Review URL

Click here to download Data Review URL http://dhsprogram.com/data/
