# Supplementary material for: Prevalence and factors associated with contraceptive use among sexually active adolescent girls in 25 sub-Saharan African countries
Source: PLoS One. 2024 Feb 28;19(2):e0297411. doi: 10.1371/journal.pone.0297411 (PMC10901330; doi:10.1371/journal.pone.0297411)
Supplement: Attachment — Submitted filename: Response to Reviewers.docx [file pone.0297411.s007.docx]

Department of Sociology

Faculty of the Social Sciences

University of Ibadan

Nigeria.

27 May 2023

Dear Editor,

PLOS ONE Journal.

We thank you and the reviewers for your thoughtful comments on the manuscript, and we have updated it to address the issues raised.

We have included a response table below that lists all of the comments raised by reviewers and how we addressed them.

We feel the work is now ready for your review and eventual publishing in PLOS ONE Journal.

Thank you very much.

Turnwait O. Michael, PhD

On behalf of all authors.

**Response Table Summary for Reviewers’ Comments**

| **Reviewer #1 Comments** | **Authors’ Actions** |
| --- | --- |
| Prevalence and determinants of contraceptive use among sexually active adolescent girls in 25 sub-Saharan African countries Previous studies - A study with a similar objective was published see link - https://contraceptionmedicine. biomedcentral.com/articles/10.1186/s40834-020-00138-1 - Why there may be argument around this, it’s important that “modern contraceptive use” is also included in the overall “contraceptive use.” | Thank you very much for identifying this study, and for bringing it to our notice. The article is referenced in our manuscript. The author, Ahinkorah (2020) focused on modern contraceptive use among adolescent girls (aged 15-19 years) and young women (aged 20-24 years). Our own study focuses primarily on sexually active adolescent girls (aged 15-19 years) who reported having been sexually active in the past four weeks before the survey. While Ahinkorah (2020) focused only on modern contraceptives, we focus on both modern and traditional contraceptive use. |
| Change all “determinants” to factors throughout the manuscript and title | Done; changed as suggested |
| This statement, “However, due to a scarcity of multi-country empirical literature”, does not show the gap in knowledge whilst this study is important. | Adjusted to now read “However, because contraceptive use among sexually active adolescents remains low and unexplored in sub-Saharan Africa, we examined…” |
|  |  |
|  |  |
| Without explicit information conviencing on why studies on modern contraceptive use among the same group in SSA is insufficient for better programmes and policies over contraceptive use, this study has not provided any knowledge gap. This argument could be the only way out of making this study unique. Why contraceptive use over modern contraceptive use despite the effective claim of modern contraceptive over other contraceptives as a whole, including “modern contraceptive”. | It is no doubt that the modern contraceptive method is considered more effective than the traditional method of contraception. We attempted to understand the use of contraceptives by adolescent girls as a whole. In addition, what makes this study stand out is the focus which is on sexually active adolescents who had been sexually active in the previous four weeks before the survey. Meaning we were not just considering adolescents as a whole, but adolescent girls that had been sexually active in the last four weeks before the survey. |
| Conclusion – not about this study examining access; why was “access” included in the conclusion? Free access might not be the issue, but other pertinent issues make recommendations in line with your study’s objective. | Adjusted to now read “It is critical for the governments and civil societies in countries with low contraceptive use to strengthen mass education on the use of contraception among sexually active adolescents, with special emphasis on the less educated, married, and adolescent girls from poor households.” |
| Introduction - Without explicit information conviencing on why studies on modern contraceptive use among the same group in SSA is insufficient for better programmes and policies over contraceptive use, this study has not provided any knowledge gap. This argument could be the only way out of making this study unique. Why contraceptive use over modern contraceptive use despite the effective claim of modern contraceptive over other contraceptives as a whole, including “modern contraceptive”. | Thanks for this clarity. Sincerely, the debate on the effectiveness of modern contraceptive methods over other methods is inarguable. We captured this in the bivariate chi-square analysis. However, our study focused primarily on examining contraceptive use as a whole among sexually active adolescent girls. Not just among adolescent girls but sexually active adolescent girls who have been sexually active in the four weeks preceding the survey. |
| Method - Well written | Thank you |
| Results - Figure 1 Remove “percentage” | Done; removed as suggested |
| Write SSA in full in all the tables | Done as suggested |
| Provide table source | Done as suggested |
| Discussion - Provide more references to other studies conducted in Africa compared to your findings. | Done as suggested; now included references to studies conducted in Ghana, Nigeria, Sierra Leone, Burkina Faso, Ethiopia, South Africa, Namibia, Chad, etc. |
| Other review - Before the conclusion, include a section on the following; 1. Implication for Research and Policy | Done as suggested; now included “Implications for Research and Policy” section |
|  |  |
| **Reviewer #2 Comments** |  |
| Thank you for the opportunity given to review that manuscript. This is an interesting study. However, I have a few comments which if the authors address will help strengthen the manuscript. | Thank you |
| 1. sub-Saharan Africa should be abbreviated in the abstract since that is the first use then “SSA” in the subsequent use | Thank you greatly. We wish to do so as suggested, however, we could not because the PLOS ONE journal submission guideline for authors instructs that there should be no abbreviation in the abstract, if possible. |
| 2. Authors should ensure that all the numbers that are not up 10 (eg: 9%, 4%) should be written in words/text | Done as suggested |
| 3. In the table 2, the authors used never in union in the presentation of the categories of marital status of the adolescents, that could give a confusing meaning/interpretation so, I suggest the author consider changing that or for example use “never married” | Done as suggested, now have “never married” |
| 4. In the table 2, the authors using two categories in the presentation of the categories of educational level of the adolescents, for example the use of below secondary is ambiguous here, because there could be people among those group of adolescents who have never had any formal education and we cannot say per se that they had below secondary level education, so I suggest new categorisation is used that is more representative | Great suggestion. Thank you very much. We have now recategorized as suggested into: no education = 0, primary education = 1, secondary education = 2, and higher education = 3 |
| 5. In the table 2, the summation of the percentage should be check to ensure they all add up exactly to 100%, as it is now some are more than 100% | Done as suggested |
| 6. The report of independent variables in unadjusted odds ratios seem to be redundant with your previous report of all independent variable being significant in the report table 2 | Thank you very much for this observation. We didn’t check well to add the significant signs. It is now been corrected. Thank you!! |
| 7. The authors report very low prevalence of contraceptive use in Chad and higher prevalence in Namibia, however, they fail to describe to the reader what Namibia is doing well (example: intervention) that might have contributed to the higher prevalence that need to be learned by other low prevalence countries | Done as suggested with examples of interventions/policy options. |
| 8. It will be helpful if the authors address grammatical errors and also the use of informal languages/English | Addressed and improved as suggested |
|  |  |
| **Reviewer #3 Comments** |  |
| Dear author(s), Congratulations for putting this together. It was worth reading and reviewing this manuscript. Please, I have made some useful comments that will improve the manuscript to be accepted for publication. Kindly consider them for your inputs. Thank you | Thank you greatly for your input. |
| Conclusions -edit | Corrected as suggested, now reads: “Conclusion” |
| Who in these countries should do this. It is always good if your recommendations are targeted towards a particular institution or institutions for action. | Done as suggested; it now reads: “It is critical for the governments and civil societies in countries with low contraceptive use to strengthen mass education on the use of contraception among sexually active adolescents, with special emphasis on the less educated, married, and adolescent girls from poor households.” |
| “While 43 births per 100 girls occurred worldwide in 2021, approximately 12 million of these births occurred in developing countries”  Dear authors, why are you mixing rates with figures? Please choose one and still to that particularly when doing comparison. | Thanks a lot for this observation. It is corrected as suggested. It now reads: “As of 2021, around 12 million births occurred among adolescents in developing countries” |
| “Adolescents who stated that they had not been sexually active in the previous four weeks were excluded from the study”  I suggest you delete this because it is repetition. Your immediate statement before this justifies your inclusion criterion. | Done as suggested; now deleted. |
| “Note: *DR = Democratic Republic; - = Not applicable”  What does this mean? You said you analysed data from 25 countries and by this table (Table 1), DR Congo is part. So, kindly explain what the Not applicable meant here. Thank you” | Thank you for your observation. The “- = Not applicable” refers to the dash (-) symbol in the body of Table 1, under the column with Year, at the point of All countries. We were not referring to DR Congo. However, to avoid further confusion for readers, we have now deleted the statement “- = Not applicable”. |
| Did you combine no education and primary education as below secondary? If yes, this is not a good choice because clearly, we expect some variation in the use of contraceptives among those who have had at least primary education. How do we see the effect of education on | Thank you greatly. We have now recategorized as suggested into: no education = 0, primary education = 1, secondary education = 2, and higher education = 3 |
| “To avoid confounding effects of explanatory variables, non-sexually active adolescent girls, as well as missing and non-responses, were dropped from the analysis”  This has already been said earlier. Please delete to avoid repetition | Done as suggested; now deleted. |
| On education, reconsider the categorisation and run the analysis again. As it stands, it does not give indepth picture. As I said earlier, you cannot combine no education with primary education to = below secondary. This is misleading and entirely unacceptable. | Thanks again for this observation. We have now recategorized as suggested into: no education, primary education, secondary education, and higher education. The analysis has also been run on this. |
| This paragraph must be revised after recording the analysis. Referring to the paragraph on education under discussion section. | Done as suggested; paragraph revised. |
|  |  |
| **Journal Requirements:** |  |
| When submitting your revision, we need you to address these additional requirements.  1. Please ensure that your manuscript meets PLOS ONE's style requirements, including those for file naming. | Done, the manuscript meets PLOS ONE's style requirements, including those for file naming. |
| 2. Please provide additional details regarding participant consent. In the ethics statement in the Methods and online submission information, please ensure that you have specified (1) whether consent was informed and (2) what type you obtained (for instance, written or verbal, and if verbal, how it was documented and witnessed). If your study included minors, state whether you obtained consent from parents or guardians. If the need for consent was waived by the ethics committee, please include this information.  If you are reporting a retrospective study of medical records or archived samples, please ensure that you have discussed whether all data were fully anonymized before you accessed them and/or whether the IRB or ethics committee waived the requirement for informed consent. If patients provided informed written consent to have data from their medical records used in research, please include this information. | Done, the ethical section now reads:  “The authors obtained written authorization from the DHS Program to use DHS datasets in this study. The DHS received ethical approval from the ICF Institutional Review Board (ICF IRB FWA00000845) in the United States, as well as from the National Health Research Ethics Committees of the countries chosen for this study. In Nigeria, for example, the National Health Research Ethics Committee of Nigeria granted ethical approval for the survey (NHREC/01/01/2007). All participants provided written informed consent. In the case of minors under the age of 18, their parents or guardians provided informed consent. There was no need for further participation consent in this study because the research used DHS (secondary) datasets. More information about DHS ethical concerns can be found at <https://goo.gl/ny8T6X>. The DHS datasets are publicly accessible at <https://dhsprogram.com/data/>.” |
| 3. Please include captions for your Supporting Information files at the end of your manuscript, and update any in-text citations to match accordingly. | Done accordingly |
