# Supplementary material for: Prevalence and factors associated with contraceptive use among sexually active adolescent girls in 25 sub-Saharan African countries
Source: PLoS One. 2024 Feb 28;19(2):e0297411. doi: 10.1371/journal.pone.0297411 (PMC10901330; doi:10.1371/journal.pone.0297411)
Supplement: Attachment — Submitted filename: Response to Reviewers.docx [file pone.0297411.s008.docx]

Department of Sociology

Faculty of the Social Sciences

University of Ibadan

Nigeria.

30 November 2023

Dear Editor,

PLOS ONE Journal.

We thank you and the reviewers for your thoughtful comments on the manuscript, and we have updated it to address the issues raised.

We have included a response table that lists all of the comments raised by reviewers and how we addressed them.

We feel the work is now ready for your review and eventual publishing in PLOS ONE Journal.

Thank you very much.

Turnwait O. Michael, PhD

On behalf of all authors.

**Response Table Summary for Reviewers and Editor’s Comments**

| **Comments** | **Authors’ Actions** |
| --- | --- |
| Note that the 2 reviewers recommend acceptance although it is suggested to copy edit the manuscript. | Thank you greatly for this recommendation. The manuscript is now copy edited as suggested. |
| In addition, as the new academic editor I have inspected the manuscript. In particular, all the mentions to "Contraceptive method knowledge" should be removed from the table and from the text. It does not make much sense to include it as a regressor and it is not included in the final model probably due to lack of balance, since no women not knowing any method was a contraceptive user. No mention is made of the reason why it was dropped in the text. It should never have been included among the potential covariates since how could a woman that declares not knowing any method report using a method? | Thank you very much for inspecting the manuscript and identifying this essential point. Contraceptive method knowledge is now removed from the table and from the text |
