# Supplementary material for: Prevalence and factors associated with contraceptive use among sexually active adolescent girls in 25 sub-Saharan African countries
Source: PLoS One. 2024 Feb 28;19(2):e0297411. doi: 10.1371/journal.pone.0297411 (PMC10901330; doi:10.1371/journal.pone.0297411)
Supplement: Attachment — Submitted filename: Response to Reviewers.docx [file pone.0297411.s009.docx]

Department of Sociology

Faculty of the Social Sciences

University of Ibadan

Nigeria.

19 December 2023

Dear Editor,

PLOS ONE Journal.

We thank you for your thoughtful comments on the manuscript, and we have updated it to address the issues raised.

We have included a response table that lists all of the comments raised and how we addressed them.

We feel the work is now ready for your review and eventual publishing in PLOS ONE Journal.

Thank you very much.

Turnwait O. Michael, PhD

On behalf of all authors.

**Response Table Summary for Reviewers and Editor’s Comments**

| **Comments** | **Authors’ Actions** |
| --- | --- |
| There was a title change from the previous version from "contraceptive use" to "contraceptives use". The international designation is "contraceptive use" so please go back to the earlier title and remove all instances (there are now many) of "contraceptives use". | Thank you for bringing this important issue to our attention and providing clarification regarding the international designation of the term "contraceptive use." We have made the necessary correction, changing 'contraceptives use' to 'contraceptive use' in the title and throughout the manuscript. |
| The description on p.6-7 on the outcome variable is unnecessarily lengthy. Since you are only considering the use of any method, just state so. You do not need to list and classify all methods. In fact you are not making any use of the question which method do you use | We have removed the unnecessarily lengthy component of the outcome variable. It now reads “The use of contraceptives by adolescent girls who were sexually active was the study’s outcome variable. Female and male sterilization, IUD, injectables, implants, pill, condom, female and male condoms, diaphragm, foam/jelly, lactational amenorrhea, rhythm, withdrawal, no methods were the options available to respondents. To create a binary outcome variable for logistic regression, the contraceptive method categories were recoded as follows: no method = 0, any method= 1. |
| L. 214: "were twice as those who could not" missing "as likely". The same problem happens in the next lines, 215, 216, 217. | We have included the “as likely” in all the affected areas. The sections now read: Adolescent girls who could request that their partners use a condom were twice as likely as those who could not. Girls who had ever been tested for HIV were twice as likely as those who had never been tested to use contraceptives. Others were more likely to use contraceptives than adolescent girls from the poorest households. Surprisingly, adolescent girls in rural areas were twice as likely as those in urban areas to use contraceptives |
| L. 233: "The less prevalence 234 of contraceptives use " should be "the lower prevalence of contraceptive use" | Corrected. It now reads: The lower prevalence of contraceptive use… |
| The interpretation of the lower prevalence of married adolescents, "Adolescent girls in SSA countries such as Nigeria, Zambia, and 265 Ethiopia are confronted by some cultural practices such as emphasizing virginity of girls, early 266 child marriage, and encouraging large family size. As a result, married adolescents are less 267 likely to use contraceptives in these countries" is missing an easier and more direct mechanism: married adolescents are less likely to use contraception because they (more of them) want to get pregnant. That is what contraceptives are for! | Corrected. This now reads: Adolescent girls in SSA countries such as Nigeria, Zambia, and Ethiopia are confronted by cultural practices such as emphasis on virginity of girls, early child marriage, and encouragement of large family sizes. As a result, married adolescents in these countries are less likely to use contraceptives as they may prioritise getting pregnant in line with the prevailing cultural norms [10, 35–37] |
| "Findings also establish that having many children can be associated with less 271 contraceptives use": Note this can't be a causal effect. In fact, you use contraception not to have children! It is rather a tell-tale sign that the particular woman does not want to use contraception or control her fertility. | Corrected. This now reads: Findings also reveal that having many children is associated with less contraceptive use. Adolescent girls who have an ideal number of 6 or more children, for example, are less likely to use contraceptives than those who have an ideal number of 0 to 2 children, as they prioritise expanding their family. |
| You should tone down this claim: "Because of their fragile age, adolescent girls who marry will undoubtedly lack the 20 347 ability to make sexual and reproductive health decisions such as asking their spouse to use a 348 condom. " You could just replace "will undoubtedly" with "might". | Corrected. This section now reads: All types of early marriage that expose young girls to early sexual debut must be prohibited by policy. This is crucial because adolescent girls who marry may lack the ability to make sexual and reproductive health decisions, such as asking their spouse to use a condom, due to their vulnerable age. |
| In addition, please remove some recommendations that do not stem from the analysis such as:  "Sexual intercourse between married adolescents and their partners is expected in most 350 societies that practise early marriage since it is regarded to be inside the marital bond [36, 37]. 351 This practice never took into account young girls' bodies and psychology, which are still 352 immature to sexual intercourse and the pregnancy repercussions" | The highlighted section is removed from the manuscript. |
| Note that no international criteria advocate universal lack of sexual activity in adolescents. You also should not say ". We advocate for sexual abstinence 354 for all adolescents".  Remember what the international consensus is that adolescents have the same SRH rights as adults, that is, "Reproductive health implies that people are able to have a satisfying and safe sex life and that they have the capability to reproduce and the freedom to decide if, when and how often to do so.". | Thanks for this insight. We have adjusted our advocacy statement to now read: We advocate that government and relevant stakeholders invest in adolescents' reproductive health and make modern contraceptives available to them in order to prevent unplanned pregnancy and its associated consequences, such as school dropout, poverty, induced abortion, and pregnancy-related health complications. |
